# Urinary CXCL9 Across the Spectrum of Kidney Allograft Inflammation

**DOI:** 10.1016/j.ekir.2026.106702

**Published:** 2026-07-11

**Authors:** Esteban Cortes Garcia, Karolien Wellekens, Claire Tinel, Stéphanie Chhun, Charlotte Debiais-Deschamps, Juliette Leon, Antoine Troger, Camille Roger, Carole Burger, Marie-Bénédicte Le Stang, Emilie Lebraud, Marie-Noelle Peraldi, Christophe Legendre, Frank Martinez, Rebecca Sberro-Soussan, Julien Zuber, Marion Rabant, Fabiola Terzi, Marie Essig, Wilfried Gwinner, Pierre Marquet, Elisabet Van Loon, Maarten Naesens, Dany Anglicheau, Olivier Aubert

**Affiliations:** 1 Université Paris Cité, Inserm UMR1151, Necker Enfants Malades Institute (INEM), Paris, France; 2 Department of Microbiology, Immunology and Transplantation, KU Leuven, Leuven, Belgium; 3 Department of Nephrology and Kidney Transplantation, Dijon University Hospital, Dijon, France; 4 Faculty of Medicine, Paris Cité University, Paris, France; 5 Institut Necker-Enfants Malades (INEM), INSERM U1151-CNRS UMR 8253, Paris, France; 6 AP-HP, Laboratory of Immunology, Necker-Enfants Malades Hospital, Paris, France; 7 Department of Kidney and Metabolic Diseases, Transplantation and Clinical Immunology, Necker Hospital, Assistance Publique - Hôpitaux de Paris, Université Paris Cité, Paris, France; 8 Department of Pathology, Necker-Enfants Malades Hospital, APHP, Université Paris Cité, Paris, France; 9 Department of Nephrology, Ambroise-Paré hospital, Assistance Publique – Hôpitaux de Paris, Paris-Saclay University, Villejuif, France; 10 Department of Nephrology and Hypertension, Hannover Medical School, Hannover, Germany; 11 INSERM U1248 Pharmacology & Transplantation, University of Limoges, Limoges University Hospital, Limoges, France; 12 Imelda Ziekenhuis, Bonheiden, Belgium

**Keywords:** BK virus nephropathy, CXCL9, kidney graft survival, rejection, urinary chemokines

## Abstract

**Background:**

Kidney allograft inflammation spans a heterogeneous spectrum of phenotypes defined by the Banff 2022 classification. We assessed whether urinary C-X-C motif chemokine ligand 9 (uCXCL9) normalized to creatinine (uCXCL9:Cr) captures inflammatory burden across this spectrum and provides incremental diagnostic utility beyond standard variables.

**Methods:**

We analyzed 3181 biopsy-urine pairs from a development cohort (1341 biopsies from 921 patients) and an independent external validation cohort (1840 biopsies from 917 patients). Associations with Banff 2022 diagnostic categories were evaluated using multinomial logistic regression with stability-based selection. Incremental performance was assessed using the Hand & Till multiclass area under the curve (AUC) and decision curve analysis.

**Results:**

uCXCL9:Cr demonstrated a graded increase across the inflammatory spectrum, with lowest levels in biopsies without specific lesions (median 4.55 ng/mmol), intermediate levels in antibody-mediated phenotypes (15.14), higher levels in acute T cell-mediated rejection (TCMR; 46.11), and the highest levels in mixed inflammatory patterns (53.18–134.05; *P* < 0.001). Levels increased proportionally with microvascular and tubulointerstitial activity scores (all *P* < 0.001). In multivariable multinomial analyses, uCXCL9:Cr was independently associated with each inflammatory category across the Banff 2022 spectrum (relative risk ratio [RRR] range: 1.68–3.44; all *P* ≤ 0.004). Addition of uCXCL9:Cr increased multiclass AUC from 0.797 to 0.831 in the development cohort and from 0.801 to 0.821 in the validation cohort (both *P* = 0.001). Incorporation of uCXCL9 was associated with a potential reduction in unnecessary biopsies in decision-curve analyses.

**Conclusion:**

uCXCL9 reflects active kidney-graft inflammation across modern Banff phenotypes and provides clinically meaningful diagnostic information beyond standard variables, supporting its potential to inform biopsy decisions and monitoring in kidney transplant recipients, pending prospective validation.

## Graphical abstract

**Figure:**
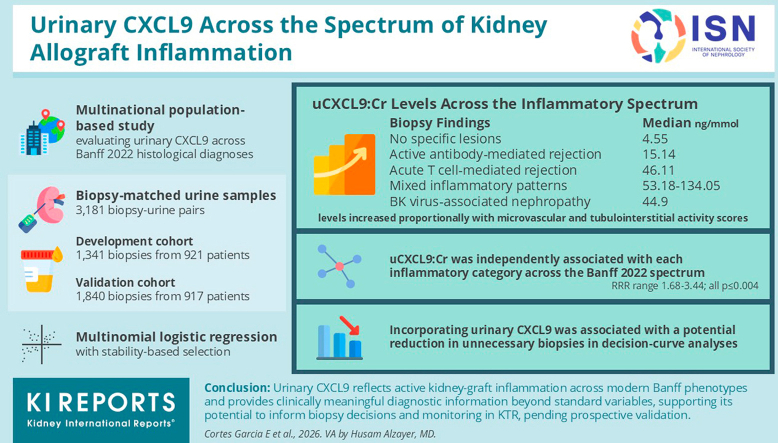

Noninvasive assessment of kidney allograft injury remains a major unmet need in transplantation. Although biopsy is the gold standard for the diagnosis of rejection, it is invasive, costly, and cannot be repeated frequently.^1^ These limitations have driven major interest in blood and urinary biomarkers to avoid unnecessary biopsies and detect early rejections.^2^, ^3^, ^4^, ^5^

uCXCL9 is induced by interferon-γ and reflects the recruitment of activated lymphocytes into the renal parenchyma. Several studies have reported increased uCXCL9 levels in clinical and subclinical rejection including T cell- or antibody-mediated rejection (AMR).^6^, ^7^, ^8^, ^9^, ^10^, ^11^ However, uCXCL9 is not specific for allograft immune injury: it also increases in BK virus associated nephropathy and other inflammatory conditions of the urinary tract.^12^, ^13^, ^14^ As a consequence, most published studies explored CXCL9 against conventional, binary diagnostic contrasts, or against the historical Banff categories, without considering the broader and more heterogeneous spectrum of lesions encountered in routine practice, including intermediate and mixed phenotypes.

This gap has become more apparent with the 2022 Banff update,^15^ which introduced 2 new diagnostic phenotypes, probable AMR and microvascular inflammation (MVI) without evidence of antibody-mediated response. These entities account for a high proportion of biopsies previously classified as “no rejection” and have been shown to carry important prognostic implications.^16^^,^^17^ To our knowledge, no study to date has examined how urinary biomarkers behave across these newly defined phenotypes or whether they can support diagnostic discrimination in this updated framework.

Previous studies have mainly relied on binary comparisons, hence they do not inform about variations of uCXCL9 across the full spectrum of inflammatory lesions encountered in clinical practice. The comprehensive understanding of how uCXCL9 relates to the contemporary Banff framework is missing, particularly in the setting of mixed or intermediate phenotypes and in conditions where alloimmune and infection-associated inflammation may coexist. Because uCXCL9 is induced across a broad range of inflammatory contexts including TCMR, MVI, mixed phenotypes, and BK virus-associated nephropathy, its interpretation should not be framed as a limitation of phenotype specificity. Rather, this broad responsiveness reflects its role as an early and sensitive marker of intragraft inflammatory activity, capturing the presence of an alloimmune or inflammatory process irrespective of its final histopathological expression and its underlying cause. In this perspective, the inability of CXCL9 to discriminate between individual Banff entities on its own does not undermine its clinical value; instead, it argues for a multicategory framework that aligns biomarker readouts with the full spectrum of contemporary Banff phenotypes and the real-world complexity of kidney allograft inflammation.

In this study, we evaluated uCXCL9 across the full spectrum of Banff 2022 histological diagnoses, including the newly defined inflammatory phenotypes, as well as intermediate and mixed disease presentations. Using biopsy-matched urine samples from development and validation cohorts, we examined the diagnostic contribution of uCXCL9 in a multicategory setting and assessed its performance in both alloimmune and BK virus-associated lesions. Our objective was to determine whether uCXCL9 provides additional diagnostic information beyond standard clinical variables within the Banff 2022 framework.

## Methods

### Study Population

In this multinational population-based study, 1838 patients from 2 centers and a consortium (Biomargin) were included with 3181 concomitant biopsy and uCXCL9:Cr evaluations. The development cohort consisted of 921 patients >18 years of age in 1 French center (Necker Hospital, Paris) with 1341 evaluations performed between 2011 and 2025. Data were retrieved from the database in November 2025. All patients provided written informed consent at the time of transplantation.

External validation was conducted on 917 patients including 597 patients from a Belgian transplantation center (Leuven hospital) (NCT01331668)^11^ and 320 from the Biomargin step 3 study^18^ with overall 1840 concomitant biopsy and uCXCL9:Cr evaluations. Data sets from the validation centers were collected as part of routine clinical practice and entered in the centers’ databases in compliance with local and national regulatory requirements and sent anonymized to our team.

### Assessment and Measurement of Urinary Chemokines

Urine samples were collected at the time of biopsy and processed within hours of collection. Samples were centrifuged (1500–3300 g, +4 °C) to pellet cells, and the cell-free supernatant stored at -80 °C until batch analysis (pellet discarded); this procedure was unchanged across centers. uCXCL9 levels were quantified using the Ella platform with Simple Plex multianalyte immunoassays (Bio-Techne, Minneapolis, MN) according to the manufacturer's instructions. Each cartridge includes a precalibrated internal standard and is read in triplicate, limiting lot-to-lot and operator variability; all centers used identical standardized protocols. All data were obtained from triplicate results for each biomarker per well. The lower limit of quantification for uCXCL9 was 39.8 pg/ml, and the mean coefficient of variation among the triplicates was less than 10% for uCXCL9. The assay has been validated by our group^19^: intra-assay and inter-assay coefficients of variation were 4.7% and 15.3%, respectively; linearity ranged from 100 to 10,000 pg/ml; the lower limit of quantification was 39.8 pg/ml in urine; and there was good correlation with reference enzyme linked immunosorbent assay (*r* = 0.61); preanalytical stability was confirmed up to 72 hours and across 5 freeze-thaw cycles. uCXCL9 concentrations were expressed in pg/ml and then normalized to uCXCL9:Cr, expressed in final units of ng/mmol.

### Clinical Data

Clinical data comprised the following: (i) demographics, including recipient’s age, sex, and transplant characteristics; (ii) laboratory results, including kidney allograft function, proteinuria, and anti-human leukocyte antigen (HLA) donor-specific antibody (DSA) specificities and levels; and (iii) allograft pathology data, including Banff lesion scores and diagnoses.^15^^,^^20^

Kidney allograft function was assessed through the estimated glomerular filtration rate (eGFR) using the Modification of Diet in Renal Disease Study equation, and proteinuria using the urine protein to creatinine ratio. Circulating DSA against HLA-A, HLA-B, HLA-Cw, HLA- DR, HLA-DQ and HLA-DP were assessed using single-antigen flow bead assays in the development cohort and according to local center practice in the validation cohorts. Although mean fluorescence intensity thresholds may have varied between centers, DSA status was consistently single antigen bead-based. The Banff 2022 categories of probable AMR and MVI without antibody-mediated evidence accommodate uncertain, weak, or negative DSA, limiting misclassification of antibody-mediated phenotypes.

### Outcome

The outcomes of interest were allograft biopsy injuries, encompassing the full spectrum of inflammatory lesions encountered in clinical practice and phenotypes defined by the Banff 2022 classification. This included AMR, TCMR, Borderline lesions, mixed rejection phenotypes, and BK virus-associated nephropathy, considered as a distinct nonalloimmune inflammatory entity. All kidney allograft biopsies were evaluated according to the Banff 2022 classification,^15^^,^^20^ including recently defined categories such as MVI DSA- and C4d- and probable AMR. These diagnostic categories were analyzed as separate outcome classes within a unified multicategory framework, allowing the comparison of alloimmune and virus-associated inflammatory phenotypes. Biopsies were read locally by experienced renal pathologists per the contemporaneous Banff criteria. To ensure consistency, diagnoses were rederived retrospectively from the recorded Banff lesion scores (g, ptc, C4d, cg, i, t, v, ci, ct, cv, ah) under Banff 2022 rather than from original labels, with reclassification where labels and lesion-based criteria diverged.

### Statistical Analysis

Continuous variables were described by using means and SDs or medians and interquartile ranges. We compared means and proportions between groups by using *t* test, analysis of variance (or Mann-Whitney test if appropriate), or the chi squared test (or Fisher’s exact test if appropriate).

We evaluated the association between the uCXCL9:Cr and each allograft diagnoses. To investigate the association of uCXCL9:Cr with rejection severity, we compared the median uCXCL9:Cr across active, chronic Banff lesion scores (ranked from 0 to 3) and 4 published indices: activity index, chronicity index, AMR/MVI index, and TCMR/TI index.^17^^,^^21^

The associations between the histological diagnoses and clinical, functional, immunological parameters and uCXCL9:Cr were assessed using multinomial logistic regression analyses after imputation of the missing data using the missForest package (version1.6.1, R Foundation, Vienna, Austria). Log-transformation was applied to the time elapsed since transplantation and uCXCL9:Cr because of their right-skewed distributions. The variables included in the multivariate model were selected using a multinomial Least Absolute Shrinkage and Selection Operator applied across 1000 bootstrap resamples, retaining predictors with a minimum selection frequency of 90%. This procedure identifies stable predictors (i.e., variables consistently selected across repeated perturbations of the dataset) thereby reducing the risk of overfitting. This stability-based selection replaces traditional *P*-value-driven stepwise procedures, which are known to be less robust and more sensitive to sampling variability.^22^, ^23^, ^24^, ^25^ Sensitivity analyses were performed using a complete-case approach, excluding observations with missing variables to assess the robustness of the multivariable model. This resulted in 94 exclusions (7.01%) in the development cohort and 242 exclusions (13.15%) in the validation cohort.

We thereafter investigated whether uCXCL9:Cr adds value to the standard of care model in detecting the histological diagnoses. To this aim, we compared the prediction performances of models with and without uCXCL9:Cr. Global model discrimination was assessed using the Hand & Till multiclass AUC (package HandTill2001 v.1.0.3.9000, R Foundation) with bias-corrected 95% CIs from 1000 bootstrap resamples, which is robust to class imbalance and provides a symmetric pairwise generalization of the AUC for multinomial outcomes.^26^ Calibration was evaluated per diagnostic class (observed vs. predicted) by grouping predicted probabilities into deciles and, within each bin, comparing the mean predicted probability with the observed event frequency. For each class of the multinomial outcome, calibration curves were generated by plotting observed versus predicted risks alongside the identity line, using predictions from both the development and validation cohorts. Internal validation relied on bootstrapping and external validation on the independent cohort. The added value of CXCL9 over the standard of care was also evaluated by means of decision curve analysis using the risk model decision analysis package (v.1.6, R Foundation).

An exploratory graft survival analysis was conducted in the development cohort. To avoid within-patient correlation, only the first biopsy with available uCXCL9:Cr measurement was retained for each patient. uCXCL9:Cr levels were dichotomized using a predefined threshold of 10 ng/mmol. Patients were classified into 4 groups according to the presence of intragraft inflammatory lesions and uCXCL9:Cr level. Death-censored graft survival was defined as the time from biopsy to graft loss, with censoring at death with a functioning graft or last follow-up. Survival curves were estimated using the Kaplan–Meier method and compared using the log-rank test.

We followed the Transparent Reporting of a Multivariable Prediction Model for Individual Prognosis or Diagnosis statement for reporting multivariable prediction model development and validation and the Standards for Reporting of Diagnostic Accuracy Studies checklist for reporting diagnostic accuracy studies.

All analyses were performed using R (version 4.3.2, R Foundation for Statistical Computing), RStudio software (version 2024.09.1+394), and STATA (version 17, StataCorp LLC, College Station, TX). Values of *P* < 0.05 were considered significant, and all tests were 2-tailed.

## Results

### Baseline Characteristics of the Kidney Allograft Recipients

The development cohort comprised a total of 921 patients corresponding to 1341 biopsies with concomitant uCXCL9:Cr assessment. The mean recipient age was 51.73 ± 15.18 years, with 572 (62.11%) males. The mean donor’s age was 55.86 ± 16.36 years. The characteristics of the recipients are summarized in Table 1. The median time between kidney transplantation and uCXCL9:Cr assessment was 0.40 (IQR: 0.24–1.10) years, with a median uCXCL9:Cr of 6.41 ng/mmol (IQR: 3.15–18.17). Antibody-mediated phenotypes occurred predominantly in for-cause biopsies (220/272, 80.9%) and at a later post-transplant time point than biopsies without specific lesions (median 0.8 years vs. 0.4 years).

**Table 1:** Baseline patient characteristics in the development and validation cohorts

Characteristics	Development cohort (*n* = 921)		External validation cohort (*n* = 917)	
	*N*		*N*	
Recipient characteristics				
Age (yrs), mean (SD)	921	51.73 (15.18)	917	54.39 (13.08)
Male sex, *n* (%)	921	572 (62.11)	917	585 (63.79)
Cause of end stage renal disease	921		917	
Glomerulopathy, *n* (%)		187 (20.30)		273 (29.77)
Polycystic kidney disease, *n* (%)		146 (15.85)		98 (10.69)
Interstitial nephritis, *n* (%)		58 (6.30)		80 (8.72)
Diabetes, *n* (%)		87 (9.45)		80 (8.72)
Vascular, *n* (%)		61 (6.62)		57 (6.22)
Other, *n* (%)		183 (19.87)		196 (21.37)
Unknown etiology, n (%)		199 (21.62)		133 (14.50)
Donor characteristics				
Age (yrs), mean (SD)	878	55.86 (16.36)	913	50.00 (14.50)
Male sex, *n* (%)	878	425 (48.41)	912	486 (53.29)
Deceased donor, n (%)	878	631 (71.87)	915	797 (87.10)
Transplant baseline characteristics				
Previous kidney transplant, n (%)	878	161 (18.34)	917	148 (16.14)
HLA-A/B/DR mismatch, mean (SD), number	878	3.96 (1.38)	870	2.98 (1.36)

HLA, human leukocyte antigen.

### Association of uCXCL9:Cr With Banff 2022 Diagnoses

**Figure 1:**
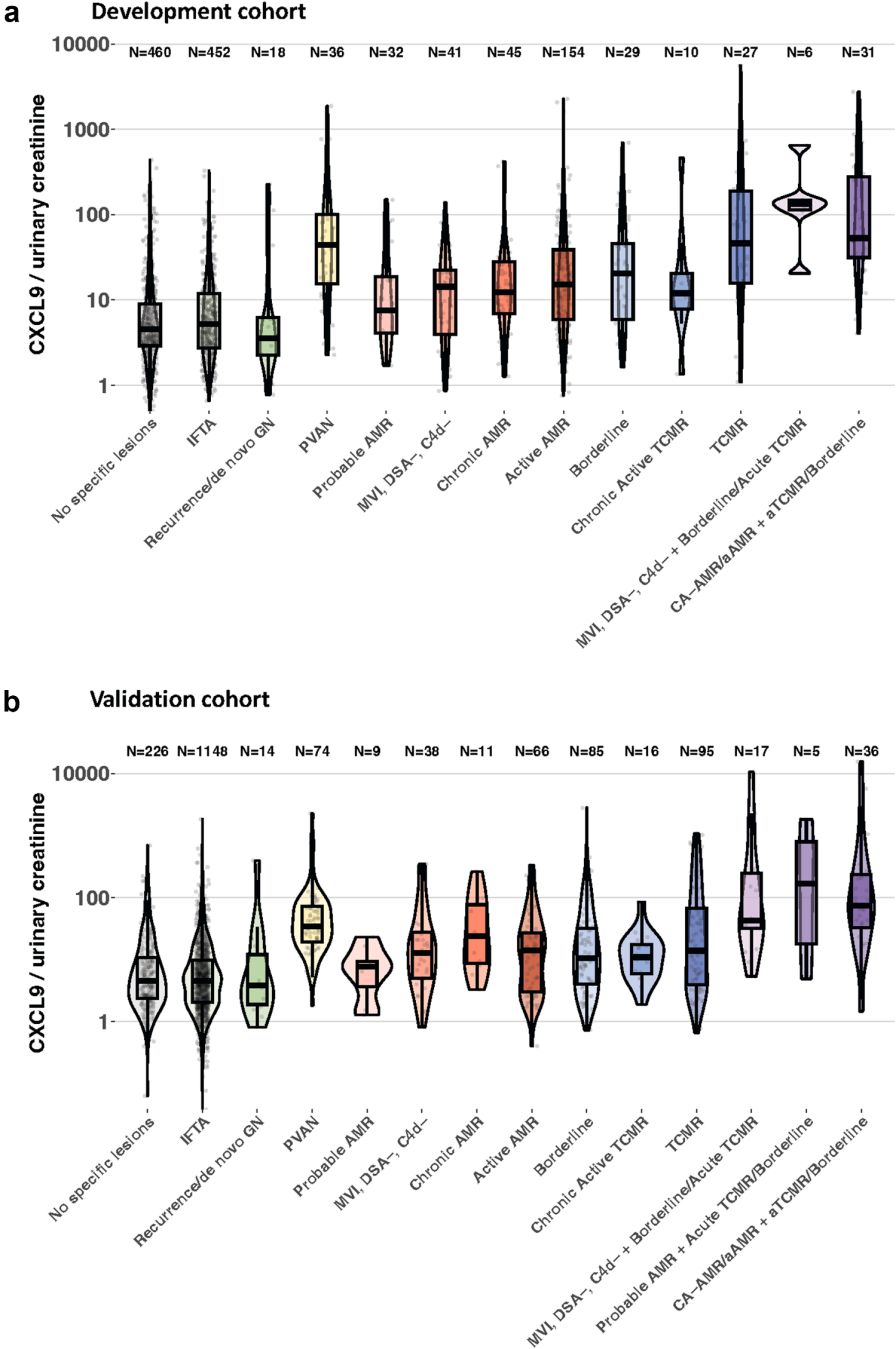
Distribution of uCXCL9 normalized to creatinine (uCXCL9:Cr) across Banff 2022 diagnostic categories in the development and validation cohorts. a (development cohort, *n* = 1341 biopsies from 921 patients) and b (validation cohort, *n* = 1840 biopsies from 917 patients) show uCXCL9:Cr across Banff 2022 categories: the upper part displays median and IQR (log scale) per category, with the number of biopsies, whose sum equals the total biopsy-urine pairs in each cohort (no missing diagnosis or uCXCL9:Cr). AMR, Antibody mediated rejection; CA-TCMR, Chronic active T-cell mediated rejection; CA-AMR, Chronic active antibody mediated rejection; DSA, Donor Specific antibody; GN, Glomerulonephritis; IFTA, Interstitial Fibrosis and Tubular Atrophy; MVI, Microvascular inflammation; PVAN, Polyomavirus associated nephropathy; TCMR, T cell mediated rejection

Among the 1341 biopsies from the development cohort, uCXCL9:Cr varied significantly across Banff diagnostic categories (Figure 1a). Biopsies with no specific lesions (median: 4.55 ng/mmol Cr, IQR: 2.92–9.00), isolated IFTA (5.27, IQR: 2.76–11.94), and recurrence and/or *de novo* glomerular diseases (3.57, IQR: 2.26–6.20) showed consistently low values.

Across AMR phenotypes, uCXCL9:Cr was elevated but heterogeneous, with a median of 15.14 (IQR: 5.94–38.80) for active AMR, 12.31 (IQR: 6.98–28.02) for chronic AMR, and 14.32 (IQR: 3.94–22.32) for MVI/DSA-/C4d- lesions. Probable AMR showed the lowest values among AMR-related categories (median: 7.58, IQR: 4.07–18.90).

TCMR phenotypes showed a broader spectrum. Acute TCMR had higher uCXCL9:Cr values (median: 46.11, IQR: 16.17–188.30), whereas chronic active TCMR had moderate levels (12.03, IQR: 7.96–20.68). Borderline changes showed intermediate values within the TCMR spectrum, with a median of 20.39 (IQR: 5.92–46.04), lower than acute TCMR but higher than nonrejection phenotypes.

Mixed-like phenotypes exhibited the highest uCXCL9:Cr values: median of 53.18 (IQR: 31.46–278.30) for chronic active-AMR/acute AMR + acute TCMR/Borderline and 134.05 (IQR: 113.54–147.63) for MVI/DSA-/C4d- + Borderline/Acute TCMR.

Outside rejection, polyomavirus associated nephropathy also showed elevated values (median: 44.49, IQR: 15.59–100.49).

### Association of uCXCL9:Cr With Inflammatory Activity

**Figure 2:**
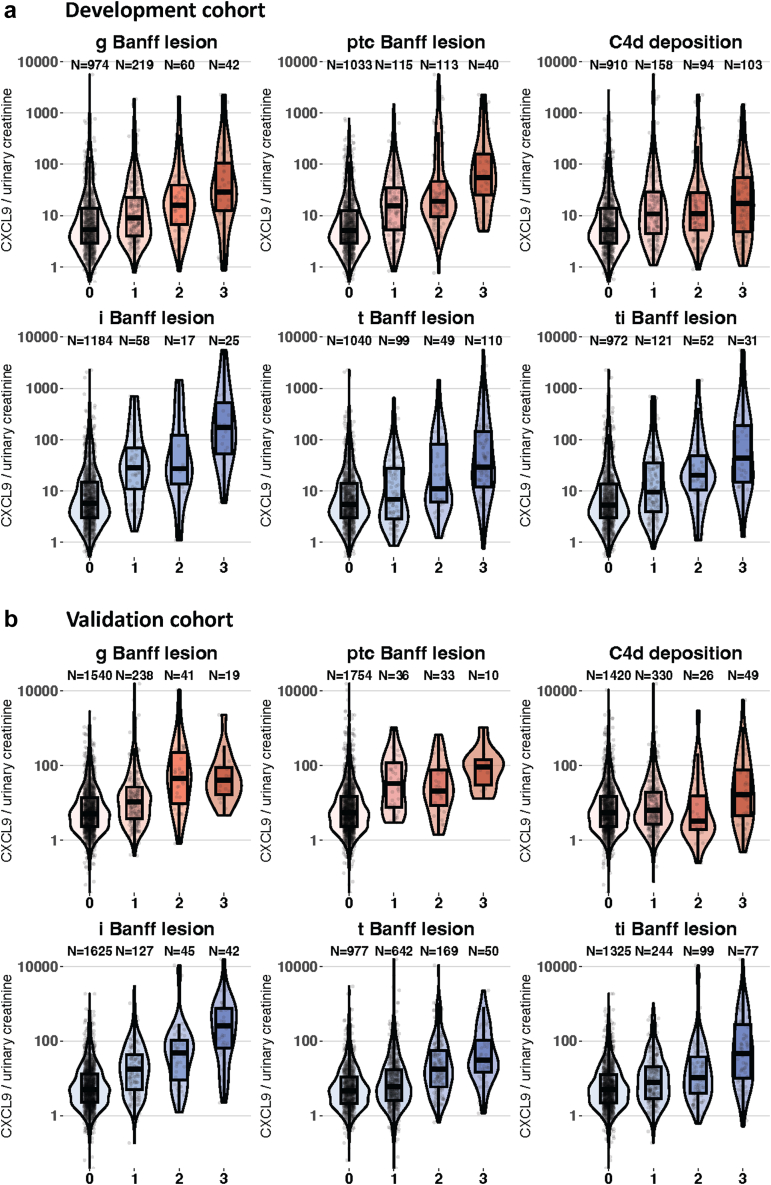
Association of uCXCL9:Cr levels with microvascular inflammation, interstitial inflammation, and tubulitis. This figure shows the distribution of uCXCL9:Cr (logarithmic y-axis) across Banff scores for glomerulitis (g), peritubular capillaritis (ptc), C4d deposition, interstitial inflammation (i), tubulitis (t), and total inflammation (ti). Each score ranges from 0 to 3, with higher scores indicating more severe lesions according to the Banff 2022 classification. (a) in the development cohort and (b) in the validation cohort. g, glomerulitis; i, interstitial inflammation; ptc, peritubular capillaritis; t, tubulitis; ti, total inflammation.

uCXCL9:Cr values increased progressively with the severity of MVI across Banff scores 0 to 3 (Figure 2a). Increasing grades of glomerulitis, peritubular capillaritis, and C4d deposition were each associated with higher uCXCL9:Cr values (all *P* < 0.0001).

Similarly, uCXCL9:Cr increased with the severity of tubulointerstitial inflammation, with graded associations observed for interstitial inflammation, tubulitis, and total interstitial inflammation scores (Figure 2a; all *P* < 0.0001).

**Figure 3:**
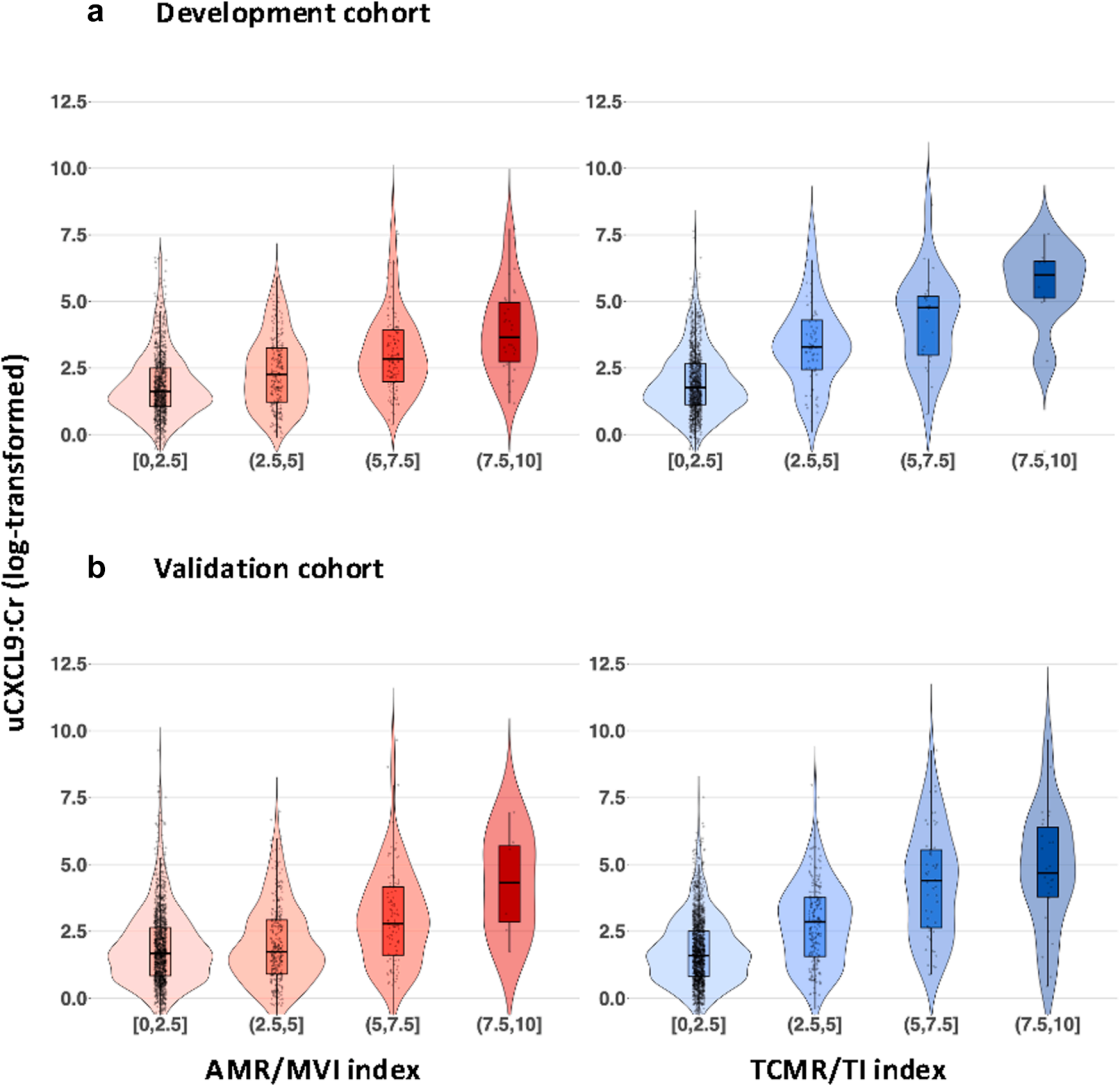
Association of uCXCL9:Cr levels with the AMR/MVI and TCMR/TI indexes. This figure shows the uCXCL9:Cr levels (logarithmic y-axis) across the AMR/MVI (red, left) and TCMR/TI (blue, right) indexes in the development cohort (a) and in the validation cohort (b). The x axis represents bins of the [0–10] ranging indexes (0–2.5, 2.5–5, 5–7.5, 7.5–10). The AMR/MVI index was defined as the weighted sum of the following Banff scores: 0.938g + 0.762ptc + 0.728cg + 2.716C4d; with C4d defined as binary variable (presence = 1 / absence = 0). The TCMR/TI index was defined as the weighted sum of the following Banff scores: 0.970i + 0.623t + 1.540v + 0.195ct. Both indexes have been rescaled in a (0–10) range as defined by Vaulet *et al.*^17^

Consistent with these findings, uCXCL9:Cr increased monotonically with higher values of the AMR/MVI and TCMR/TI composite indices defined by Vaulet *et al.*^17^ (Figure 3), which integrate microvascular and tubulointerstitial lesion severity into continuous inflammatory scores ranging from 0 to 10. Both gradients were observed in the development cohort (Panel A) and reproduced in the validation cohort (Panel B). uCXCL9:Cr also increased with higher values of the Banff activity index (Supplementary Figure S1).

In contrast, uCXCL9:Cr values did not vary meaningfully across chronic Banff lesions, including interstitial fibrosis, tubular atrophy, vascular fibrous intimal thickening, arteriolar hyalinosis, or across the chronicity index (ci + ct + cv + 2cg; range 0–10), with overlapping distributions across lesion grades (Supplementary Figures S2A and S3A).

### Independent and Added Value of uCXCL9:Cr to Detect Allograft Inflammation

Associations between clinical, functional, and immunological variables and kidney biopsy diagnoses were assessed using multinomial logistic regression with the following 5 mutually exclusive outcomes: AMR-spectrum microvascular phenotypes, TCMR-spectrum tubulointerstitial inflammation, mixed microvascular and T cell–mediated inflammation, BK virus nephropathy, and biopsies without specific lesions (reference category). Univariate analyses are reported in Supplementary Table S1.

Variable selection for the multivariable model was performed in the development cohort using a multinomial least absolute shrinkage and selection operator with bootstrap-based stability selection (1000 resamples), retaining predictors selected in ≥ 90% of samples (Supplementary Figure S4). The final multivariable multinomial model was then refitted using the selected predictors to obtain relative risk ratios and confidence intervals.

In multivariable analyses, uCXCL9:Cr was independently associated with all inflammatory diagnostic categories, including AMR-spectrum lesions (RRR 1.677 [95% CI: 1.477–1.904]; *P* < 0.001), TCMR-spectrum lesions (RRR 2.291 [1.899–2.763]; *P* < 0.001), mixed phenotypes (RRR 3.443 [2.661–4.454]; *P* < 0.001), and polyomavirus associated nephropathy (RRR 2.200 [1.527–3.170]; *P* < 0.001). No other predictor demonstrated consistent associations across all inflammatory phenotypes. Full multivariable estimates are reported in Table 2.

**Table 2:** Determinants of histologic diagnoses in the development cohort: multivariable multinomial analysis

Variables			N obs.	AMR/MVI spectrum			TCMR/TI spectrum			Mixed phenotypes			Viral nephropathy		
				*N* (%)	RRR (95% CI)	*P*	*N* (%)	RRR (95% CI)	*P*	*N* (%)	RRR (95% CI)	*P*	*N* (%)	RRR (95% CI)	*P*
Transplant baseline characteristics	Previous kidney transplant	No	1106	190 (17.18)	1	-	58 (5.24)	1	-	26 (2.35)	1	-	30 (2.71)	1	-
		Yes	235	82 (34.89)	2.444 (1.682–3.552)	< 0.001	8 (3.40)	0.772 (0.348–1.710)	0.523	11 (4.68)	2.275 (1.002–5.168)	0.050	6 (2.55)	1.400 (0.423–4.638)	0.582
	HLA A/B/DR mismatches (per 1 mismatch increment)		1341	272 (20.28)	1.226 (1.082–1.390)	0.001	66 (4.92)	1.178 (0.954–1.453)	0.127	37 (2.76)	1.147 (0.842–1.563)	0.384	36 (2.68)	1.264 (0.880–1.817)	0.205
Time of biopsy	Time from transplant to biopsy (log-transformed) (per 1-log increment)		1341	272 (20.28)	1.338 (1.209–1.480)	< 0.001	66 (4.92)	1.033 (0.875–1.218)	0.704	37 (2.76)	1.194 (0.981–1.454)	0.078	36 (2.68)	1.695 (1.032–2.782)	0.037
Functional parameters	eGFR (mL/min per 1.73 m^2^)		1341	272 (20.28)	0.981 (0.972–0.990)	< 0.001	66 (4.92)	0.990 (0.976–1.005)	0.202	37 (2.76)	0.982 (0.961–1.004)	0.105	36 (2.68)	0.985 (0.958–1.013)	0.291
	Leukocyturia	No	983	209 (21.26)	1	-	47 (4.78)	1	-	20 (2.03)	1	-	25 (2.54)	1	-
		Yes	358	63 (17.60)	0.616 (0.425–0.893)	0.011	19 (5.31)	0.659 (0.360–1.206)	0.176	17 (4.75)	1.028 (0.478–2.209)	0.944	11 (3.07)	0.519 (0.178–1.513)	0.230
	BK viremia	No	1252	262 (20.93)	1	-	61 (4.87)	1	-	36 (2.88)	1	-	2 (0.16)	1	-
		Yes	89	10 (11.24)	0.502 (0.228–1.103)	0.086	5 (5.62)	0.837 (0.299–2.344)	0.735	1 (1.12)	0.213 (0.026–1.766)	0.152	34 (38.20)	200.414 (43.569–921.877)	< 0.001
Immunological parameters at the time of biopsy	Anti-HLA donor-specific antibody	No	867	98 (11.30)	1	-	44 (5.07)	1	-	17 (1.96)	1	-	24 (2.77)	1	**-**
		Yes	474	174 (36.71)	4.708 (3.439–6.446)	< 0.001	22 (4.64)	1.442 (0.823–2.525)	0.200	20 (4.22)	3.790 (1.788–8.035)	0.001	12 (2.53)	1.228 (0.476–3.164)	0.671
	uCXCL9:Cr (log-transformed)		1341	272 (20.28)	1.677 (1.477–1.904)	< 0.001	66 (4.92)	2.291 (1.899–2.763)	< 0.001	37 (2.76)	3.443 (2.661–4.454)	< 0.001	36 (2.68)	2.200 (1.527–3.170)	< 0.001

AMR, antibody mediated rejection; DSA, donor-specific antibody; eGFR, estimated glomerular filtration rate; MVI, microvascular inflammation; RRR, relative risk ratio; TCMR, T cell mediated rejection.

**Figure 4:**
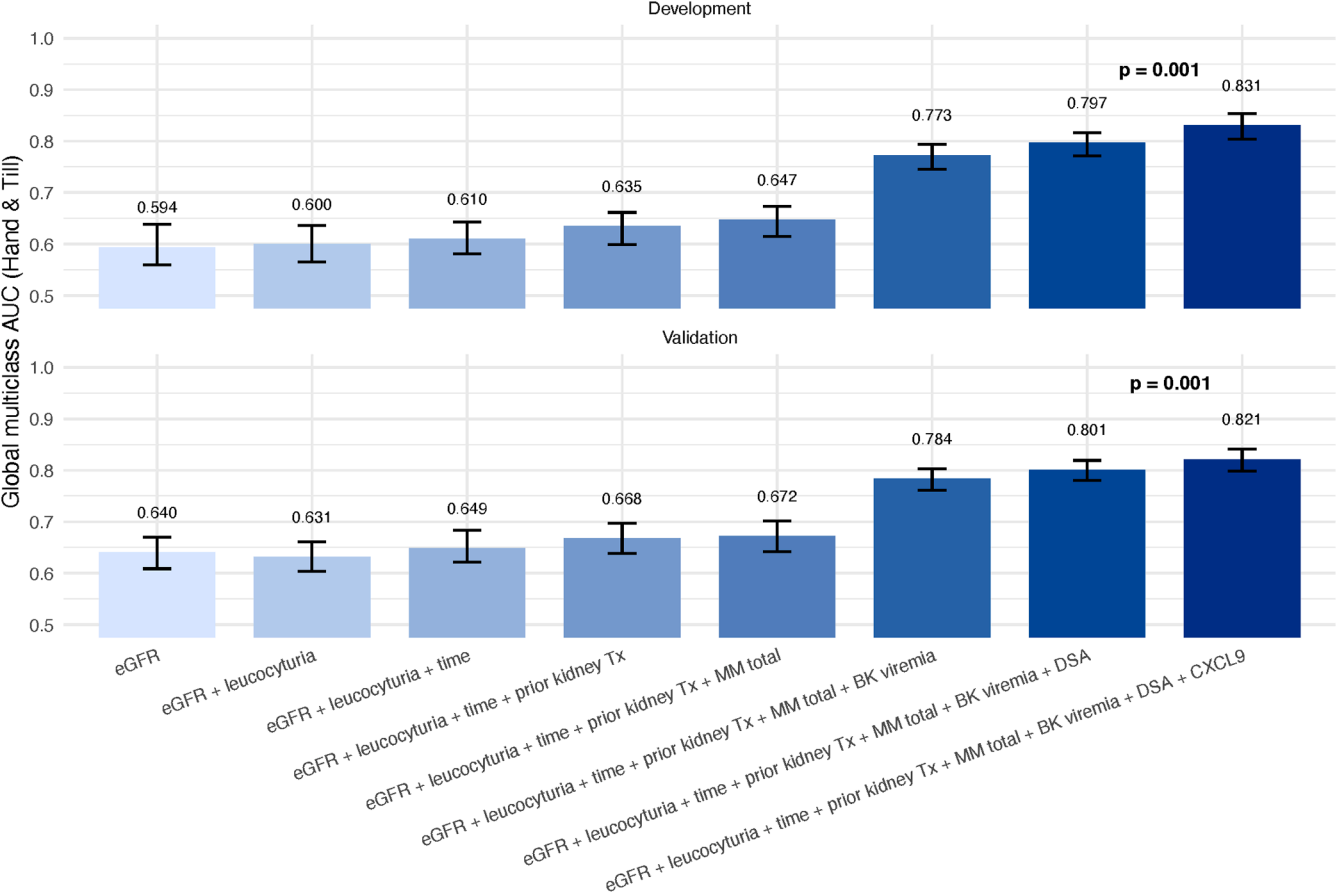
Incremental multiclass diagnostic performance of stepwise clinical models with uCXCL9:Cr in the development and in the validation cohort. Stepwise construction of multinomial models starting from eGFR alone, with sequential addition of clinical and immunological variables, culminating in uCXCL9:Cr. Model discrimination was quantified using the Hand and Till multiclass AUC (bars), with bias-corrected 95% confidence intervals (error bars). Discrimination improved progressively across models, with a marked and statistically significant increase following the inclusion of uCXCL9:Cr compared with the immediately preceding model (*P* = 0.001). Panel a corresponds to the development cohort and panel b to the validation cohort. DSA, Donor Specific Antibodies; HLA, human leukocyte antigen.

The incremental diagnostic value of uCXCL9:Cr was evaluated by comparing multivariable models with and without its inclusion. In stepwise model construction starting from eGFR alone, discrimination improved progressively, with the Hand and Till multiclass AUC increasing from 0.594 (bias-corrected 95% CI: 0.560–0.638) to 0.831 (bias-corrected 95% CI: 0.804–0.853) following the addition of uCXCL9:Cr (*P* = 0.001; Figure 4a). Pairwise comparisons further confirmed significant improvements in multiclass discrimination when uCXCL9:Cr was added to single-variable baseline models (all *P* < 0.001; Supplementary Figure S5A).

**Figure 5:**
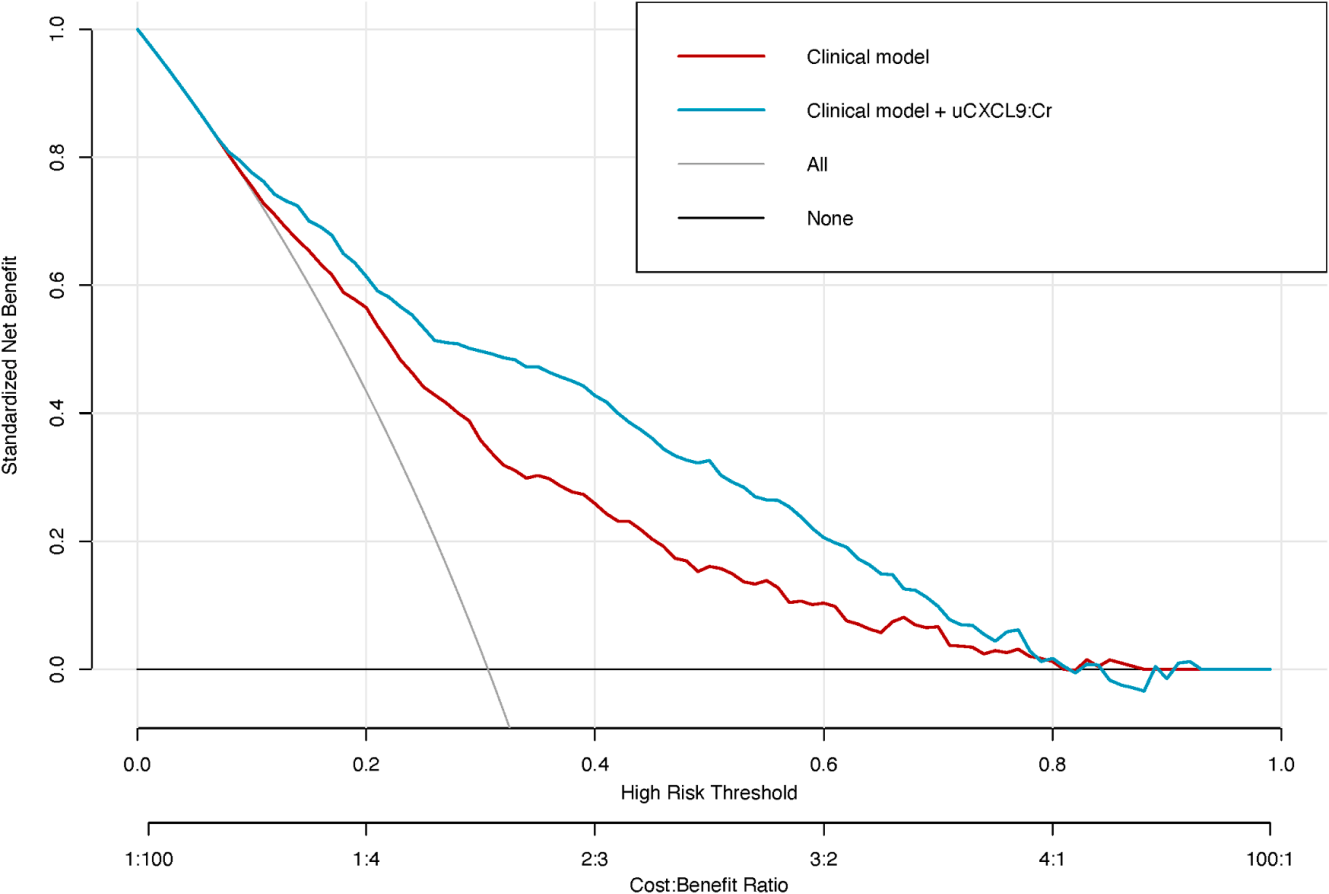
Decision curve analysis of clinical models with and without uCXCL9:Cr in the development cohort. Decision curve analysis showing the net benefit of the clinical model alone versus the clinical model including creatinine-corrected uCXCL9 across threshold probabilities in the development cohort. The model incorporating CXCL9 provides a consistently higher net benefit between 0.10 and 0.70, the range in which biopsy decisions are typically uncertain. In this interval, CXCL9 improves identification of biopsies likely to show intragraft activity while reducing unnecessary procedures compared with the clinical model alone, as well as the “biopsy-all” and “biopsy-none” strategies.

The model incorporating uCXCL9:Cr demonstrated appropriate calibration across the full range of predicted risks (Supplementary Figure S6A). Decision curve analysis showed a consistently higher net benefit for the uCXCL9-augmented model compared with the clinical model alone, as well as the “biopsy-all” and “biopsy-none” strategies, across threshold probabilities of 0.10 to 0.70 (Figure 5). A threshold of 0.32 maximized avoidance of unnecessary biopsies, corresponding to a 10.9% reduction (Supplementary Figure S7).

Stratification by biopsy indication revealed a context-dependent incremental value of uCXCL9:Cr. In the development cohort, addition of uCXCL9:Cr improved multiclass discrimination in both protocol biopsies (AUC 0.833–0.904; *P* = 0.006) and for-cause biopsies (AUC 0.789–0.838; *P* < 0.001). Among protocol biopsies, the prevalence of active inflammatory lesions increased progressively with higher uCXCL9:Cr levels. In the development cohort, active lesions were present in 22.2%, 32.2%, 36.4% and 40.4% of protocol biopsies with uCXCL9:Cr values ≥ 5, ≥ 10, ≥ 15 and ≥ 20 ng/mmol, respectively.

### Graft Survival According to Biopsy Phenotype and uCXCL9:Cr

**Figure 6:**
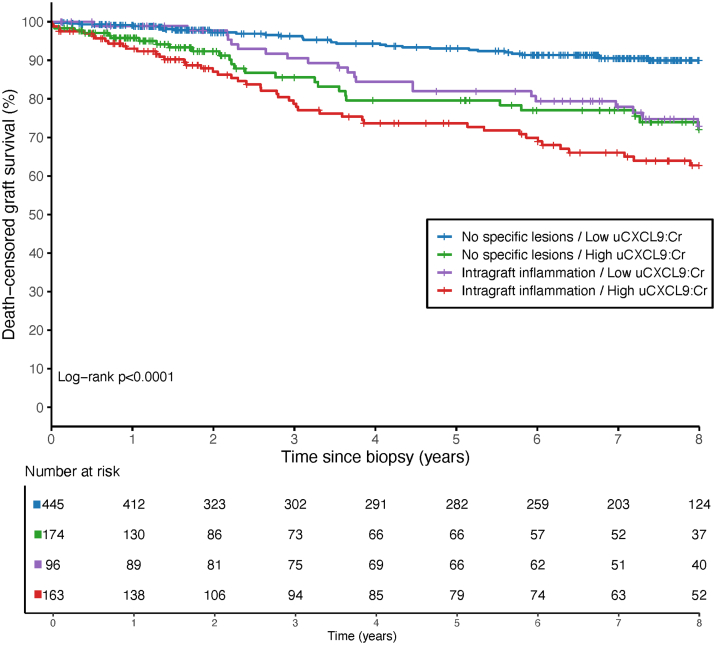
Kaplan-Meier curves of death-censored graft survival according to biopsy phenotype and uCXCL9:Cr level in the development cohort. Patients were classified into 4 groups based on the presence or absence of intragraft inflammatory lesions and uCXCL9:Cr level, using a predefined threshold of uCXCL9:Cr >10 to define high CXCL9. One biopsy per patient was considered, retaining the first biopsy with available uCXCL9:Cr measurement. Survival curves were compared using the log-rank test.

Using a predefined threshold of uCXCL9:Cr > 10 ng/mmol to define high CXCL9 levels, death-censored graft survival differed significantly across the 4 biopsy phenotypes in the development cohort (log-rank *P* < 0.0001). At 8 years post-biopsy, graft survival was highest among patients without specific histological lesions and low uCXCL9:Cr (90.0%; 95% CI: 86.7–93.4). In contrast, graft survival was markedly lower and comparable among patients without specific lesions but high uCXCL9:Cr (72.0%; 95% CI: 62.9–82.4) and those with intragraft inflammation and low uCXCL9:Cr (72.9%; 95% CI: 63.5–83.8). The poorest graft survival was observed in patients with intragraft inflammation and high uCXCL9:Cr (62.7%; 95% CI: 54.5–72.2; Figure 6).

### External Validation

The validation cohort comprised 917 patients with 1840 paired biopsy-urine evaluations (Table 1). The median time from transplantation to uCXCL9:Cr assessment was 0.67 years (IQR: 0.24–1.10), and the median uCXCL9:Cr was 5.67 ng/mmol Cr (IQR: 2.45–16.25).

The distribution of uCXCL9:Cr across Banff 2022 diagnostic categories closely mirrored that observed in the development cohort (Figure 1b), with low values in biopsies without inflammatory lesions, intermediate levels across AMR-spectrum phenotypes (lowest in probable AMR), broader distributions across TCMR phenotypes, and the highest levels in mixed microvascular and tubulointerstitial inflammation and in polyomavirus associated nephropathy.

Associations between uCXCL9:Cr and histological activity were reproduced in the validation cohort, with stepwise increases according to glomerulitis, peritubular capillaritis, interstitial inflammation, and tubulitis severity (all *P* < 0.001; Figure 2b), as well as across the AMR/MVI and TCMR/TI indexes (Figure 3b). uCXCL9:Cr showed no meaningful variation across chronic lesion scores or the chronicity index (Supplementary Figures S2B-S3B-S8).

In multivariable multinomial analyses, uCXCL9:Cr remained independently associated with all inflammatory diagnostic categories, including AMR-spectrum lesions, TCMR-spectrum lesions, mixed phenotypes, and polyomavirus associated nephropathy, after adjustment for standard clinical and immunological variables (Supplementary Table S2).

When models were refitted using the same prespecified predictors as in the development cohort, the addition of uCXCL9:Cr significantly improved multicategory discrimination, with the Hand and Till AUC increasing from 0.640 to 0.821 (*P* < 0.001; Figure 4b). Consistent improvements were observed across pairwise comparisons with single-variable baseline models (Supplementary Figure S5B). Model calibration remained satisfactory (Supplementary Figure S6B), and decision curve analysis confirmed a consistent incremental net benefit across threshold probabilities of 0.10 to 0.70 (Supplementary Figure S9).

### uCXCL9:Cr Diagnostic Performance Across Predefined Thresholds and Continuous Evaluation

We further explored the diagnostic performance of uCXCL9:Cr used as a standalone biomarker across a range of predefined thresholds, using standard threshold-based metrics including sensitivity, specificity, predictive values, and global accuracy indices. These analyses provide descriptive context for the operating characteristics of uCXCL9:Cr across predefined thresholds.

In the development cohort, a uCXCL9:Cr threshold of 10 ng/mmoL was associated with a relatively balanced sensitivity–specificity profile (0.657 and 0.737, respectively), alongside favorable Matthews correlation coefficient (0.373) and Youden index (0.393), while maintaining a high negative predictive value (0.829; Supplementary Table S3). As expected, lower thresholds were associated with higher sensitivity at the expense of specificity, whereas higher thresholds increased specificity with a concomitant reduction in sensitivity.

Similar operating characteristics were observed in the validation cohort. Using the same predefined uCXCL9:Cr threshold of 10 yielded comparable sensitivity and specificity (0.648 and 0.749, respectively), with consistent Matthews correlation coefficient (0.359), Youden index (0.398), and negative predictive value (0.867; Supplementary Table S4).

### Sensitivity Analyses

To assess the robustness of the primary findings to missing data, complete-case sensitivity analyses were performed in both cohorts. Complete cases accounted for 1247 observations (93.0%) in the derivation cohort and 1598 observations (86.8%) in the validation cohort. In complete-case multivariable multinomial analyses, uCXCL9:Cr remained independently associated with all inflammatory diagnostic categories.

In the derivation cohort, uCXCL9:Cr was associated with AMR-spectrum microvascular phenotypes (RRR 1.636 [95% CI: 1.428–1.874]; *P* < 0.001), TCMR-spectrum tubulointerstitial inflammation (RRR 2.296 [1.878–2.808]; *P* < 0.001), mixed inflammatory phenotypes (RRR 3.996 [2.951–5.412]; *P* < 0.001), and BK virus-associated nephropathy (RRR 1.993 [1.354–2.933]; *P* < 0.001). Comparable associations were observed in the validation cohort for AMR-spectrum phenotypes (RRR 2.107 [1.691–2.625]; *P* < 0.001), TCMR-spectrum inflammation (RRR 1.514 [1.226–1.870]; *P* < 0.001), mixed phenotypes (RRR 2.912 [2.168–3.913]; *P* < 0.001), and BK virus–associated nephropathy (RRR 2.744 [1.908–3.945]; *P* < 0.001) (Supplementary Tables S5 and S6).

Consistent with these associations, inclusion of uCXCL9:Cr in complete-case models was associated with a significant improvement in multiclass discrimination. The AUC increased from 0.805 (bias-corrected 95% CI: 0.781–0.827) to 0.849 (0.819–0.874; *P* < 0.001) in the derivation cohort and from 0.805 (0.784–0.826) to 0.827 (0.805–0.849; *P* <0.001) in the validation cohort (Supplementary Figure S10).

Sensitivity analyses using absolute uCXCL9 concentrations instead of creatinine-normalized values yielded similar results. In the development cohort, the addition of absolute uCXCL9 increased the Hand-and-Till AUC from 0.798 (95% CI: 0.772–0.817) to 0.828 (0.804–0.849; *P* < 0.01).

Decision-curve analysis stratified by indication showed context-dependent utility: at a 0.30 threshold, net benefit was 0.034 (for-cause) and 0.030 (protocol) in the development cohort, and 0.047 and 0.019 in the validation cohort; greatest in for-cause biopsies, with protocol biopsies still showing reduced unnecessary procedures.

## Discussion

In this multicenter study, we evaluated uCXCL9 across the full spectrum of histological phenotypes defined by the Banff 2022 classification, including recently introduced entities, such as probable AMR and MVI without evidence of antibody-mediated response. Using 3181 biopsy-urine pairs from development and independent external validation cohorts, we demonstrate that uCXCL9:Cr increases proportionally with the severity and extent of active histological inflammation across contemporary Banff categories.

uCXCL9:Cr was found to behave as a continuous marker of inflammatory burden rather than as a phenotype-specific signal. uCXCL9 was independently associated with all major inflammatory diagnostic groups, including AMR-spectrum lesions, TCMR, mixed phenotypes, and BK virus-associated nephropathy. Moreover, uCXCL9:Cr increased proportionally with the severity of both microvascular and tubulointerstitial Banff lesion scores, as well as with composite inflammatory indices integrating these lesions. In contrast, no meaningful association was observed with chronic lesions or chronicity indices. Together, these results support the interpretation of CXCL9 as a marker reflecting active intragraft inflammation rather than cumulative or irreversible tissue damage.

This behavior is highly consistent with recent conceptual advances in transplant pathology. The Banff 2022 update emphasized that kidney allograft inflammation exists along a continuum rather than as discrete categories, particularly for microvascular and tubulointerstitial lesions.^15^ In line with this, Vaulet *et al.*^17^ proposed continuous indices capturing the phenotypic spectrum of rejection and demonstrated graded associations between inflammatory burden and graft outcomes. Similarly, Sablik *et al.*^16^ showed that MVI, including phenotypes previously classified as “no rejection,” is associated with adverse graft outcomes. The graded increase of uCXCL9:Cr across Banff lesion scores and composite inflammatory indices observed in our study mirrors this biological and pathological continuum, suggesting that uCXCL9 captures the same underlying inflammatory signal described by these histological frameworks.

Importantly, we confirmed that CXCL9 is not specific for rejection. Elevated levels were also observed in BK virus-associated nephropathy, consistent with previous reports and with the known biology of CXCL9 as an interferon-γ-inducible chemokine produced by tubular epithelial and endothelial cells during immune activation.^9^^,^^12^^,^^14^ Rather than representing a limitation, this lack of specificity reinforces the role of CXCL9 as a global indicator of intrarenal inflammatory activity. In clinical practice, uCXCL9 is best interpreted within a stepwise diagnostic framework rather than as a stand-alone test: elevated levels signal active intragraft inflammation without specifying its mechanism and should prompt etiological work-up (BK viremia, DSA, eGFR trajectory, proteinuria, and urinary tract infection) before a biopsy decision, whereas low levels in the absence of graft dysfunction identify recipients in whom biopsy may reasonably be deferred. This use of uCXCL9 as a sensitive readout of inflammatory activity feeding an etiology-oriented algorithm is consistent with the European Society for Organ Transplantation 2023 consensus recommendation on noninvasive monitoring of immune quiescence.^27^

Our findings extend previous work on uCXCL9: Clinical Trials in Organ Transplantation-01^28^ first showed it discriminated acute rejection and that low 6-month levels identified low-risk recipients, and Clinical Trials in Organ Transplantation-04^29^ extended this to urinary-cell mRNA, with later refinements.^7^^,^^10^^,^^11^ Our study is the largest external validation to date (3181 pairs, 3 centers, 2 countries), the first across the Banff 2022 spectrum (including probable AMR and MVI without antibody-mediated evidence), and the first to use a multicategory rather than binary framework.

Our results reconcile with the recent KTD-Innov study^6^: its more limited utility reflects a binary rejection-versus-no-rejection design in which CXCL9-inducing entities (BK nephropathy, urinary tract infection) populated the control group, lowering apparent specificity. Our multicategory framework instead makes explicit that uCXCL9 reflects intragraft inflammation irrespective of cause. The 2 studies nonetheless converge: CXCL9 improved discrimination (AUROC 0.75 to 0.78 in KTD-Innov; 0.797 to 0.831 here) with a high negative predictive value, supporting its role as a marker of immune quiescence rather than a rejection-specific test.

uCXCL9 should be interpreted within the broader landscape of noninvasive biomarkers for kidney allograft injury, which include donor-derived cell-free DNA (reflecting graft injury and cellular turnover), blood and tissue gene-expression signatures (e.g., kidney solid organ response test, TruGraf, the Molecular Microscope) integrating broader immune pathways, and urinary mRNA profiles.^30^, ^31^, ^32^, ^33^, ^34^ Among these, uCXCL9 specifically reflects local intragraft interferon-γ-driven inflammatory activity and offers practical advantages, such as a fully automated immunoassay with a 90-minute turnaround, low cost, and direct measurement on a noninvasively obtained fluid. These properties position it as a readily implementable indicator of inflammatory activity that complements, rather than competes with, existing strategies and could form the first step of a tiered noninvasive monitoring algorithm.

A multinomial modeling strategy was necessary to reflect the inherent complexity of contemporary Banff 2022 diagnoses. Binary diagnostic contrasts oversimplify biomarker behavior and fail to capture clinically relevant gradients across intermediate and mixed inflammatory phenotypes. By modeling all Banff categories simultaneously, we provide a realistic representation of uCXCL9:Cr performance across routine biopsy diagnoses. Incorporation of uCXCL9:Cr into clinical models resulted in consistent improvements in multicategory discrimination, with preserved calibration and demonstrable net clinical benefit on decision curve analysis. Within this framework of overlapping inflammatory phenotypes, the observed gains in discrimination are particularly informative in diagnostically uncertain settings, where incremental biomarker information has the greatest clinical relevance.

From a translational perspective, uCXCL9 could feed a risk-based biopsy-decision algorithm: in stable recipients a low value (below a threshold from a model integrating eGFR, DSA status, BK viremia, and proteinuria) would support deferring biopsy, whereas elevated values would prompt etiological work-up and, if indicated, biopsy-feasible at the bedside on a 90-minute automated platform at predefined visits.

An exploratory, unadjusted survival analysis was concordant: graft survival was poorest with both inflammation and high uCXCL9:Cr, best with neither, and intermediate for discordant profiles, suggesting uCXCL9 captures inflammation not always evident on a single biopsy. As an unadjusted, exploratory analysis it is hypothesis-generating only and should not be read as prognostic; adjusted, time-updated models are needed to confirm it.

Several limitations should be acknowledged. First, this analysis was based on biopsy-matched urine samples obtained at a single time point and therefore does not address longitudinal dynamics or the predictive value of uCXCL9 over time. Second, although the cohort was large, several less frequent Banff 2022 subgroups (MVI without antibody-mediated evidence, probable AMR, mixed patterns) were small, limiting the precision of subgroup-specific estimates. Multinomial models with stability selection borrow information across categories and limit unstable estimation, but subgroup findings should be regarded as exploratory and confirmed in larger cohorts. Third, despite retrospective harmonization of diagnoses based on lesion scores, residual interobserver variability in histological interpretation across centers cannot be fully excluded. The retrospective application of the Banff 2022 classification across a long inclusion period (2011–2025) entails potential era-related limitations. Certain diagnostic entities, such as chronic-active TCMR, were not explicitly defined in earlier Banff iterations and may not be fully reconstructible from historical data, despite our use of detailed lesion scores rather than legacy diagnostic labels. Some residual misclassification of these entities therefore cannot be excluded. However, because our primary analyses are anchored on continuous lesion scores and composite inflammatory indices—which have remained stable across Banff iterations—the central finding that uCXCL9 reflects intragraft inflammatory activity should be robust to era-related shifts in diagnostic taxonomy. Fourth, residual confounding cannot be fully excluded, particularly in BK virus-associated nephropathy, where viral kinetics and treatment modifications may influence both biomarker levels and histological findings. Fifth, uCXCL9 was evaluated as a single biomarker, and its performance in combination with other noninvasive tools was not assessed in this study. Last, decision-curve analysis was used to explore potential clinical utility. It is sensitive to model calibration and outcome prevalence, and a model-based projection of avoided biopsies does not itself demonstrate benefit; any biopsy reduction must be confirmed in prospective interventional studies before clinical use.

In conclusion, uCXCL9 is independently associated with inflammatory phenotypes across the Banff 2022 spectrum and reflects the severity of active intrarenal inflammation. As a noninvasive marker of inflammatory burden, uCXCL9 could inform biopsy decisions, pending prospective interventional validation.

## Study Approval

Each patient included in either the development or the validation cohort provided written informed consent to be included in the French national registry agency (Agence de la Biomédecine) databases CRISTAL and DIVAT. The DIVAT and CRISTAL database networks have been approved by the National French Commission for Bioinformatics, Data, and Patient Liberty: DIVAT: CNIL, registration number: 1016618, validated 8 June 2004; and CRISTAL: CNIL, registration number: 363505, validated 3 April 1996.

## Disclosure

All the authors declared no competing interests.

## Supplementary Material

Supplementary file (PDF) **Figure S1.** Association of uCXCL9:Cr with the activity index. **Figure S2.** Association of uCXCL9:Cr with interstitial fibrosis, tubular atrophy, vascular fibrous intimal thickening and arteriolar hyalinosis. **Figure S3.** Association of uCXCL9:Cr with the chronicity index. **Figure S4.** Bootstrap multinomial LASSO feature selection. **Figure S5.** Consistent incremental multiclass discrimination conferred by uCXCL9:Cr across single-variable models in the development and in the validation cohort. **Figure S6.** Multiclass calibration curves in the development and validation cohorts. **Figure S7.** Decision curve–derived reduction in unnecessary and total biopsies with uCXCL9:Cr. **Figure S8.** AMR/MVI index distributions for high vs low chronicity index in the validation cohort. **Figure S9.** Decision curve analysis of clinical model with and without uCXCL9:Cr in the validation cohort. **Figure S10.** Performances of models with or without uCXCL9:Cr in predicting kidney allograft biopsy lesions (complete case analysis). **Table S1.** Determinants of histologic diagnoses in the development cohort: univariate multinomial analysis. **Table S2.** Determinants of histologic diagnoses in the validation cohort: multivariable multinomial analysis. **Table S3.** Diagnostic performance metrics of uCXCL9:Cr ratio across predefined thresholds in the development cohort. **Table S4.** Diagnostic performance metrics of uCXCL9:Cr ratio across predefined thresholds in the validation cohort. **Table S5.** Determinants of histologic diagnoses in the development cohort: multivariable multinomial analysis (complete case analysis). **Table S6.** Determinants of histologic diagnoses in the validation cohort: multivariable multinomial analysis (complete case analysis). **STARD 2015. TRIPOD Checklist**.

## Footnotes

Supplementary file (PDF)
**Figure S1.** Association of uCXCL9:Cr with the activity index. **Figure S2.** Association of uCXCL9:Cr with interstitial fibrosis, tubular atrophy, vascular fibrous intimal thickening and arteriolar hyalinosis. **Figure S3.** Association of uCXCL9:Cr with the chronicity index. **Figure S4.** Bootstrap multinomial LASSO feature selection. **Figure S5.** Consistent incremental multiclass discrimination conferred by uCXCL9:Cr across single-variable models in the development and in the validation cohort. **Figure S6.** Multiclass calibration curves in the development and validation cohorts. **Figure S7.** Decision curve–derived reduction in unnecessary and total biopsies with uCXCL9:Cr. **Figure S8.** AMR/MVI index distributions for high vs low chronicity index in the validation cohort. **Figure S9.** Decision curve analysis of clinical model with and without uCXCL9:Cr in the validation cohort. **Figure S10.** Performances of models with or without uCXCL9:Cr in predicting kidney allograft biopsy lesions (complete case analysis). **Table S1.** Determinants of histologic diagnoses in the development cohort: univariate multinomial analysis. **Table S2.** Determinants of histologic diagnoses in the validation cohort: multivariable multinomial analysis. **Table S3.** Diagnostic performance metrics of uCXCL9:Cr ratio across predefined thresholds in the development cohort. **Table S4.** Diagnostic performance metrics of uCXCL9:Cr ratio across predefined thresholds in the validation cohort. **Table S5.** Determinants of histologic diagnoses in the development cohort: multivariable multinomial analysis (complete case analysis). **Table S6.** Determinants of histologic diagnoses in the validation cohort: multivariable multinomial analysis (complete case analysis). **STARD 2015** **Checklist****. TRIPOD Checklist.**
