# Supplementary material for: Urinary CXCL9 Across the Spectrum of Kidney Allograft Inflammation
Source: Kidney Int Rep. 2026 Jul 11;11(10):106702. doi: 10.1016/j.ekir.2026.106702 (PMC13486795; doi:10.1016/j.ekir.2026.106702)
Supplement: Supplementary file 1 [file mmc1.pdf]

Supplementary appendix

1. SUPPLEMENTARY TABLES

Supplementary Table 1: Determinants of histologic diagnoses in the development cohort: univariate multinomial analysis

Supplementary Table 2: Determinants of histologic diagnoses in the validation cohort: multivariable multinomial analysis

Supplementary Table 3: Diagnostic performance metrics of uCXCL9:Cr ratio across predefined thresholds in the development cohort

Supplementary Table 4: Diagnostic performance metrics of uCXCL9:Cr ratio across predefined thresholds in the validation cohort

Supplementary Table 5: Determinants of histologic diagnoses in the development cohort: multivariable multinomial analysis (complete case analysis)

Supplementary Table 6: Determinants of histologic diagnoses in the validation cohort: multivariable multinomial analysis (complete case analysis)

2. SUPPLEMENTARY FIGURES

Supplementary Figure 1: Association of uCXCL9:Cr with the activity index

Supplementary Figure 2: Association of uCXCL9:Cr with interstitial fibrosis, tubular atrophy, vascular fibrous intimal thickening and arteriolar hyalinosis

Supplementary Figure 3: Association of uCXCL9:Cr with the chronicity index

Supplementary Figure 4: Bootstrap multinomial LASSO feature selection

Supplementary Figure 5: Consistent incremental multiclass discrimination conferred by uCXCL9:Cr across single-variable models in the development and in the validation cohort.

Supplementary Figure 6: Multiclass calibration curves in the development and validation cohorts

Supplementary Figure 7: Decision curve-derived reduction in unnecessary biopsies with uCXCL9:Cr

Supplementary Figure 8: AMR/MVI index distributions for high vs low chronicity index in the validation cohort

Supplementary Figure 9: Decision curve analysis of clinical model with and without uCXCL9:Cr in the validation cohort

Supplementary Figure 10: Performances of models with or without uCXCL9:Cr in predicting kidney allograft biopsy lesions (complete case analysis)

Supplementary Table 1: Determinants of histologic diagnoses in the development cohort: univariate multinomial analysis

			N obs.	N MVI (%)	RRR (95%IC)	p	N TCMR (%)	RRR (95% IC),	p	N MVI / TCMR (%)	RRR (95% IC)	p	N BKV (%)	RRR (95% IC)	p
**Recipient baseline characteristics**	**Age** (per 1-yr increment)		1,341	272 (20.28)	0.988 (0.979–0.997)	**0.007**	66 (4.92)	0.999 (0.982–1.016)	0.881	37 (2.76)	0.962 (0.940–0.984)	**< 0.001**	36 (2.68)	1.002 (0.980–1.025)	0.859
	**Sex**	**Female**	506	111 (21.94)	1	-	31 (6.13)	1	-	12 (2.37)	1	-	19 (3.75)	1	-
		**Male**	835	161 (19.28)	0.809 (0.614–1.067)	0.133	35 (4.19)	0.630 (0.381–1.040)	0.071	25 (2.99)	1.162 (0.576–2.343)	0.675	17 (2.04)	0.499 (0.256–0.973)	**0.041**
**Donor baseline characteristics**	**Age** (per 1-yr increment)		1,341	272 (20.28)	0.992 (0.983–1.000)	**0.049**	66 (4.92)	0.994 (0.979–1.009)	0.431	37 (2.76)	0.983 (0.964–1.003)	0.094	36 (2.68)	0.998 (0.978–1.019)	0.866
	**Sex**	**Female**	697	142 (20.37)	1	-	30 (4.30)	1	-	24 (3.44)	1	-	20 (2.87)	1	-
		**Male**	644	130 (20.19)	0.989 (0.755–1.296)	0.937	36 (5.59)	1.297 (0.785–2.141)	0.310	13 (2.02)	0.585 (0.294–1.164)	0.127	16 (2.48)	0.864 (0.442–1.689)	0.670
	**Living donor**	**No**	938	191 (20.36)	1	-	49 (5.22)	1	-	22 (2.35)	1	-	22 (2.35)	1	-
		**Yes**	403	81 (20.10)	0.995 (0.740–1.336)	0.972	17 (4.22)	0.814 (0.461–1.438)	0.478	15 (3.72)	1.599 (0.817–3.128)	0.171	14 (3.47)	1.493 (0.753–2.961)	0.251
**Transplant baseline characteristics**	**Prior kidney transplant**	**No**	1,106	190 (17.18)	1	-	58 (5.24)	1	-	26 (2.35)	1	-	30 (2.71)	1	-
		**Yes**	235	82 (34.89)	2.704 (1.965–3.721)	**< 0.001**	8 (3.40)	0.864 (0.403–1.852)	0.707	11 (4.68)	2.651 (1.278–5.496)	**0.009**	6 (2.55)	1.253 (0.512–3.071)	0.621
	**Cold ischemia time**	**<12 hours**	757	143 (18.89)	1	-	31 (4.10)	1	-	18 (2.38)	1	-	20 (2.64)	1	-
		**12-24 hours**	412	91 (22.09)	1.282 (0.949–1.732)	0.105	29 (7.04)	1.885 (1.113–3.193)	**0.018**	14 (3.40)	1.567 (0.768–3.199)	0.217	12 (2.91)	1.209 (0.582–2.510)	0.611
		**≥24 hours**	172	38 (22.09)	1.210 (0.804–1.822)	0.361	6 (3.49)	0.881 (0.360–2.160)	0.783	5 (2.91)	1.265 (0.461–3.475)	0.648	4 (2.33)	0.911 (0.306–2.714)	0.867
	**HLA A/B/DR mismatches (per 1 mismatch more)**		1,341	272 (20.28)	1.190 (1.073–1.321)	**0.001**	66 (4.92)	1.250 (1.028–1.520)	**0.025**	37 (2.76)	1.223 (0.947–1.579)	0.124	36 (2.68)	1.302 (0.999–1.695)	0.051
**Time of biopsy**	**Log(Time from transplant to biopsy)** (per 1-year increment)		1,341	272 (20.28)	1.269 (1.156–1.394)	**< 0.001**	66 (4.92)	0.911 (0.768–1.081)	0.287	37 (2.76)	1.038 (0.830–1.299)	0.744	36 (2.68)	1.275 (1.016–1.599)	**0.036**
**Functional parameters**	**eGFR (mL/min/1.73 m^2)**		1,341	272 (20.28)	0.978 (0.971–0.986)	**< 0.001**	66 (4.92)	0.977 (0.963–0.991)	**0.002**	37 (2.76)	0.952 (0.932–0.973)	**< 0.001**	36 (2.68)	0.976 (0.957–0.995)	**0.014**

	**Log(Proteinuria) (g/g)**		1,341	272 (20.28)	1.515 (1.348–1.701)	**< 0.001**	66 (4.92)	1.186 (0.952–1.477)	0.128	37 (2.76)	1.783 (1.383–2.300)	**< 0.001**	36 (2.68)	1.051 (0.774–1.428)	0.750
	**Leucocyturia**	**No**	983	209 (21.26)	1	-	47 (4.78)	1	-	20 (2.03)	1	-	25 (2.54)	1	-
		**Yes**	358	63 (17.60)	0.829 (0.604–1.138)	0.246	19 (5.31)	1.112 (0.640–1.932)	0.706	17 (4.75)	2.335 (1.203–4.529)	**0.012**	11 (3.07)	1.210 (0.587–2.496)	0.605
	**BK viremia**	**No**	1,252	262 (20.93)	1	-	61 (4.87)	1	-	36 (2.88)	1	**-**	2 (0.16)	1	-
		**Yes**	89	10 (11.24)	0.872 (0.429–1.770)	0.705	5 (5.61)	1.873 (0.712–4.922)	0.203	1 (1.12)	0.635 (0.085–4.749)	0.658	34 (38.20)	388.38 (90.04–1,675.19)	**< 0.001**
**Immunological parameters at the time of biopsy**	**Anti-HLA donor-specific antibody**	**No**	867	98 (11.30)	1	-	44 (5.07)	1	-	17 (1.96)	1	-	24 (2.77)	1	-
		**Yes**	474	174 (36.71)	4.937 (3.704–6.579)	**< 0.001**	22 (4.64)	1.390 (0.817–2.367)	0.225	20 (4.22)	3.272 (1.686–6.347)	**< 0.001**	12 (2.53)	1.390 (0.685–2.822)	0.362
	**Log(CXCL9 / Creatinine)**		1,341	272 (20.28)	1.741 (1.557–1.946)	**< 0.001**	66 (4.92)	2.406 (2.013–2.876)	**< 0.001**	37 (2.76)	3.660 (2.898–4.623)	**< 0.001**	36 (2.68)	2.793 (2.231–3.496)	**< 0.001**

RRR: relative risk ratio; MVI: AMR-spectrum microvascular phenotypes; TCMR: TCMR-spectrum tubulointerstitial inflammation; MVI/TCMR: mixed microvascular and T cell–mediated inflammation; BKV: viral nephropathy

Supplementary Table 2: Determinants of histologic diagnoses in the validation cohort: multivariable multinomial analysis

			N obs.	N MVI (%)	RRR (95%IC)	p	N TCMR (%)	RRR (95% IC),	p	N MVI / TCMR (%)	RRR (95% IC)	p	N BKV (%)	RRR (95% IC)	p
**Transplant baseline characteristics**	**Prior kidney transplant**	**No**	1,576	94 (5.96)	1	-	178 (11.29)	1	-	44 (2.79)	1	-	63 (4.00)	1	-
		**Yes**	264	30 (11.36)	1.737 (1.073–2.811)	**0.025**	18 (6.82)	0.664 (0.392–1.126)	0.128	14 (5.30)	1.281 (0.609–2.692)	0.513	11 (4.16)	0.689 (0.314–1.515)	0.355
	**HLA A/B/DR mismatches (per 1 mismatch more)**		1,840	124 (6.74)	0.932 (0.798–1.088)	0.371	196 (10.65)	0.972 (0.857–1.103)	0.660	58 (3.15)	0.680 (0.539–0.859)	**0.001**	74 (4.02)	0.877 (0.705–1.091)	0.239
**Time of biopsy**	**Log(Time from transplant to biopsy)** (per 1-year increment)		1,840	124 (6.74)	1.269 (1.090–1.476)	**0.002**	196 (10.65)	0.789 (0.699–0.890)	**< 0.001**	58 (3.15)	0.843 (0.694–1.025)	0.087	74 (4.02)	0.923 (0.718–1.188)	0.536
**Functional parameters**	**eGFR (mL/min/1.73 m^2)**		1,840	124 (6.74)	0.994 (0.983–1.004)	0.240	196 (10.65)	0.979 (0.969–0.988)	**< 0.001**	58 (3.15)	0.968 (0.950–0.987)	**0.001**	74 (4.02)	0.984 (0.969-1.000)	**0.050**
	**Leukocyturia**	**No**	1,264	84 (6.65)	1	-	118 (9.34)	1	-	25 (1.98)	1	-	52 (4.11)	1	-
		**Yes**	576	40 (6.94)	1.334 (0.869–2.047)	0.188	78 (13.54)	1.027 (0.729–1.449)	0.877	33 (5.73)	1.726 (0.906–3.289)	0.097	22 (3.82)	0.618 (0.329–1.159)	0.133
	**BK viremia**	**No**	1,534	107 (6.98)	1	-	172 (11.21)	1	-	55 (3.59)	1	-	4 (0.26)	1	-
		**Yes**	306	17 (5.56)	0.849 (0.488–1.479)	0.564	24 (7.84)	0.911 (0.565–1.468)	0.702	3 (0.98)	0.380 (0.114–1.266)	0.115	70 (22.88)	101.275 (35.809–286.423)	**< 0.001**
**Immunological parameters at the time of biopsy**	**Anti-HLA donor-specific antibody**	**No**	1,728	97 (5.61)	1	-	190 (11.00)	1	-	44 (2.55)	1	-	69 (3.99)	1	-
		**Yes**	112	27 (24.11)	4.111 (2.366–7.142)	**< 0.001**	6 (5.36)	0.661 (0.275–1.586)	0.353	14 (12.50)	5.413 (2.487–11.783)	**< 0.001**	5 (4.46)	1.892 (0.557–6.421)	0.307
	**Log(CXCL9 / Creatinine)**		1,840	124 (6.74)	2.130 (1.746–2.598)	**< 0.001**	196 (10.65)	1.614 (1.336–1.949)	**< 0.001**	58 (3.15)	2.847 (2.195–3.692)	**< 0.001**	74 (4.02)	2.574 (1.854–3.573)	**< 0.001**

RRR: relative risk ratio; MVI: AMR-spectrum microvascular phenotypes; TCMR: TCMR-spectrum tubulointerstitial inflammation; MVI/TCMR: mixed microvascular and T cell–mediated inflammation; BKV: viral nephropathy

Supplementary Table 3: Diagnostic performance metrics of uCXCL9:Cr ratio across predefined thresholds in the development cohort
Diagnostic performance metrics of the urinary CXCL9:creatinine ratio (uCXCL9:Cr) were calculated across predefined thresholds using untransformed values. The outcome of interest was biopsy-proven graft activity, defined as any diagnosis other than *No specific lesions*. Metrics include measures of discrimination, predictive values, and overall classification performance. Thresholds are expressed in the original uCXCL9:Cr scale.

uCXCL9:Cr threshold	TP	TN	FP	FN	N	Prevalence	Test pos rate	Sensitivity	Specificity	PPV	NPV	Accuracy	Balanced accuracy	F1	MCC	Youden	LR pos	LR neg	Brier
5	335	484	446	76	1341	0.306	0.582	0.815	0.520	0.429	0.864	0.611	0.668	0.562	0.314	0.336	1.700	0.355	0.389
**10**	**270**	**685**	**245**	**141**	**1341**	**0.306**	**0.384**	**0.657**	**0.737**	**0.524**	**0.829**	**0.712**	**0.697**	**0.583**	**0.373**	**0.393**	**2.494**	**0.466**	**0.288**
15	229	768	162	182	1341	0.306	0.292	0.557	0.826	0.586	0.808	0.743	0.691	0.571	0.389	0.383	3.199	0.536	0.257
20	195	813	117	216	1341	0.306	0.233	0.474	0.874	0.625	0.790	0.752	0.674	0.539	0.380	0.349	3.771	0.601	0.248
25	167	831	99	244	1341	0.306	0.198	0.406	0.894	0.628	0.773	0.744	0.650	0.493	0.347	0.300	3.817	0.664	0.256
30	147	847	83	264	1341	0.306	0.172	0.358	0.911	0.639	0.762	0.741	0.634	0.459	0.328	0.268	4.008	0.705	0.259
35	134	862	68	277	1341	0.306	0.151	0.326	0.927	0.663	0.757	0.743	0.626	0.437	0.326	0.253	4.459	0.727	0.257
40	126	867	63	285	1341	0.306	0.141	0.307	0.932	0.667	0.753	0.740	0.619	0.420	0.316	0.239	4.526	0.744	0.260
45	116	872	58	295	1341	0.306	0.130	0.282	0.938	0.667	0.747	0.737	0.610	0.397	0.302	0.220	4.526	0.766	0.263
50	105	878	52	306	1341	0.306	0.117	0.255	0.944	0.669	0.742	0.733	0.600	0.370	0.286	0.200	4.569	0.789	0.267

TP: True positives; TN: True negatives; FP: false positives; FN: False negatives; PPV: Positive predictive value; NPV: Negative predictive value; MCC: Matthews correlation coefficient; LR pos: Positive likelihood ratio; LR neg: Negative likelihood ratio

Supplementary Table 4: Diagnostic performance metrics of uCXCL9:Cr ratio across predefined thresholds in the validation cohort

Diagnostic performance metrics of the urinary CXCL9:creatinine ratio (uCXCL9:Cr) were calculated across predefined thresholds using untransformed values. The outcome of interest was biopsy-proven graft activity, defined as any diagnosis other than *No specific lesions*. Metrics include measures of discrimination, predictive values, and overall classification performance. Thresholds are expressed in the original uCXCL9:Cr scale.

uCXCL9:Cr threshold	TP	TN	FP	FN	N	Prevalence	Test pos rate	Sensitivity	Specificity	PPV	NPV	Accuracy	Balanced accuracy	F1	MCC	Youden	LR pos	LR neg	Brier
5	351	753	635	101	1840	0.246	0.536	0.777	0.543	0.356	0.882	0.600	0.660	0.488	0.275	0.319	1.697	0.412	0.400
**10**	**293**	**1040**	**348**	**159**	**1840**	**0.246**	**0.348**	**0.648**	**0.749**	**0.457**	**0.867**	**0.724**	**0.699**	**0.536**	**0.359**	**0.398**	**2.585**	**0.469**	**0.276**
15	251	1149	239	201	1840	0.246	0.266	0.555	0.828	0.512	0.851	0.761	0.692	0.533	0.373	0.383	3.225	0.537	0.239
20	220	1222	166	232	1840	0.246	0.210	0.487	0.880	0.570	0.840	0.784	0.684	0.525	0.388	0.367	4.070	0.583	0.216
25	195	1260	128	257	1840	0.246	0.176	0.431	0.908	0.604	0.831	0.791	0.670	0.503	0.384	0.339	4.678	0.626	0.209
30	175	1291	97	277	1840	0.246	0.148	0.387	0.930	0.643	0.823	0.797	0.659	0.483	0.385	0.317	5.540	0.659	0.203
35	161	1308	80	291	1840	0.246	0.131	0.356	0.942	0.668	0.818	0.798	0.649	0.465	0.381	0.299	6.180	0.683	0.202
40	148	1314	74	304	1840	0.246	0.121	0.327	0.947	0.667	0.812	0.795	0.637	0.439	0.362	0.274	6.142	0.710	0.205
45	136	1325	63	316	1840	0.246	0.108	0.301	0.955	0.683	0.807	0.794	0.628	0.418	0.354	0.255	6.629	0.732	0.206
50	130	1331	57	322	1840	0.246	0.102	0.288	0.959	0.695	0.805	0.794	0.623	0.407	0.351	0.247	7.004	0.743	0.206

TP: True positives; TN: True negatives; FP: false positives; FN: False negatives; PPV: Positive predictive value; NPV: Negative predictive value; MCC: Matthews correlation coefficient; LR pos: Positive likelihood ratio; LR neg: Negative likelihood ratio

Supplementary Table 5: Determinants of histologic diagnoses in the development cohort: multivariable multinomial analysis (complete case analysis)

			N obs.	N MVI (%)	RRR (95%IC)	p	N TCMR (%)	RRR (95% IC),	p	N MVI / TCMR (%)	RRR (95% IC)	p	N BKV (%)	RRR (95% IC)	p
**Transplant baseline characteristics**	**Prior kidney transplant**	**No**	1,022	161 (15.75)	1	-	53 (5.19)	1	-	21 (2.05)	1	-	27 (2.64)	1	-
		**Yes**	225	79 (35.11)	2.727 (1.858–4.001)	**< 0.001**	8 (3.56)	0.826 (0.370–1.842)	0.640	10 (4.44)	2.594 (1.054–6.385)	**0.038**	6 (2.67)	1.774 (0.512–6.144)	0.366
	**HLA A/B/DR mismatches (per 1 mismatch more)**		1,247	240 (19.25)	1.254 (1.103–1.427)	**0.001**	61 (4.89)	1.201 (0.970–1.487)	0.093	31 (2.49)	1.299 (0.924–1.826)	0.133	33 (2.65)	1.245 (0.858–1.807)	0.248
**Time of biopsy**	**Log(Time from transplant to biopsy)** (per 1-year increment)		1,247	240 (19.25)	1.269 (1.134–1.420)	**< 0.001**	61 (4.89)	1.020 (0.847–1.228)	0.835	31 (2.49)	1.027 (0.815–1.294)	0.819	33 (2.65)	1.793 (1.005–3.200)	**0.048**
**Functional parameters**	**eGFR (mL/min/1.73 m^2)**		1,247	240 (19.25)	0.981 (0.972–0.991)	**< 0.001**	61 (4.89)	0.994 (0.979–1.009)	0.432	31 (2.49)	0.982 (0.958–1.005)	0.128	33 (2.65)	0.988 (0.960–1.017)	0.417
	**Leukocyturia**	**No**	905	180 (18.89)	1	-	42 (4.64)	1	-	17 (1.88)	1	-	22 (2.43)	1	-
		**Yes**	342	60 (17.54)	0.675 (0.462–0.986)	**0.042**	19 (5.56)	0.755 (0.410–1.390)	0.366	14 (4.09)	0.857 (0.361–2.034)	0.726	11 (3.22)	0.664 (0.225–1.963)	0.459
	**BK viremia**	**No**	1,162	230 (19.79)	1	-	56 (4.82)	1	-	31 (2.67)	1	-	1 (0.09)	1	-
		**Yes**	80	7 (8.75)	0.535 (0.239–1.197)	0.128	5 (6.25)	0.745 (0.261–2.129)	0.582	0 (0.00)	0.000 (0.000–0.000)	-	32 (40.00)	336.908 (42.712–2,657.481)	**< 0.001**
**Immunological parameters at the time of biopsy**	**Anti-HLA donor-specific antibody**	**No**	807	85 (10.53)	1	-	41 (5.08)	1	-	15 (1.86)	1	-	22 (2.73)	1	-
		**Yes**	440	155 (35.23)	4.616 (3.323–6.411)	**< 0.001**	20 (4.55)	1.265 (0.707–2.262)	0.429	16 (3.64)	2.772 (1.205–6.377)	**0.016**	11 (2.50)	1.247 (0.458–3.394)	0.666
	**Log(CXCL9 / Creatinine)**		1,247	240 (19.25)	1.636 (1.428–1.874)	**< 0.001**	61 (4.89)	2.296 (1.878–2.808)	**< 0.001**	31 (2.49)	3.996 (2.951–5.412)	**< 0.001**	33 (2.65)	1.993 (1.354–2.933)	**< 0.001**

RRR: relative risk ratio; MVI: AMR-spectrum microvascular phenotypes; TCMR: TCMR-spectrum tubulointerstitial inflammation; MVI/TCMR: mixed microvascular and T cell–mediated inflammation; BKV: viral nephropathy

Supplementary Table 6: Determinants of histologic diagnoses in the validation cohort: multivariable multinomial analysis (complete case analysis)

			N obs.	N MVI (%)	RRR (95%IC)	p	N TCMR (%)	RRR (95% IC),	p	N MVI / TCMR (%)	RRR (95% IC)	p	N BKV (%)	RRR (95% IC)	p
**Transplant baseline characteristics**	**Prior kidney transplant**	**No**	1,374	83 (6.04)	1	-	161 (11.72)	1	-	35 (2.55)	1	-	62 (4.51)	1	-
		**Yes**	224	25 (11.16)	1.574 (0.933–2.655)	0.089	12 (3.36)	0.505 (0.270–0.947)	**0.033**	11 (4.91)	1.510 (0.666–3.424)	0.324	10 (4.46)	0.642 (0.285–1.448)	0.285
	**HLA A/B/DR mismatches (per 1 mismatch more)**		1,598	108 (6.76)	0.938 (0.797–1.104)	0.443	173 (10.83)	1.014 (0.888–1.158)	0.836	46 (2.88)	0.709 (0.547–0.920)	**0.010**	72 (4.51)	0.854 (0.684–1.066)	0.163
**Time of biopsy**	**Log(Time from transplant to biopsy)** (per 1-year increment)		1,598	108 (6.76)	1.295 (1.089–1.541)	**0.003**	173 (10.83)	0.772 (0.673–0.884)	**< 0.001**	46 (2.88)	0.875 (0.695–1.102)	0.257	72 (4.51)	0.891 (0.685–1.160)	0.393
**Functional parameters**	**eGFR (mL/min/1.73 m^2)**		1,598	108 (6.76)	0.996 (0.985–1.008)	0.532	173 (10.83)	0.975 (0.964–0.985)	**< 0.001**	46 (2.88)	0.960 (0.938–0.983)	**0.001**	72 (4.51)	0.984 (0.968–1.000)	0.057
	**Leukocyturia**	**No**	1,089	75 (6.89)	1	-	104 (9.55)	1	-	18 (1.65)	1	-	50 (4.59)	1	-
		**Yes**	509	33 (6.48)	1.154 (0.726–1.834)	0.545	69 (13.56)	0.923 (0.637–1.338)	0.673	28 (5.50)	1.864 (0.899–3.864)	0.094	22 (4.32)	0.613 (0.321–1.170)	0.138
	**BK viremia**	**No**	1,317	93 (7.06)	1	-	149 (11.31)	1	-	43 (3.26)	1	-	3 (0.23)	1	-
		**Yes**	281	15 (5.34)	0.816 (0.452–1.475)	0.501	24 (8.54)	1.122 (0.685–1.838)	0.647	3 (1.06)	0.496 (0.146–1.692)	0.263	69 (24.56)	133.185 (40.360–439.505)	**< 0.001**
**Immunological parameters at the time of biopsy**	**Anti-HLA donor-specific antibody**	**No**	1,504	83 (5.52)	1	-	171 (11.37)	1	-	36 (2.39)	1	-	67 (4.45)	1	-
		**Yes**	94	25 (26.60)	4.958 (2.779–8.844)	**< 0.001**	2 (2.13)	0.247 (0.059–1.041)	0.057	10 (10.64)	4.671 (1.907–11.438)	**0.001**	5 (5.32)	1.858 (0.520–6.641)	0.340
	**Log(CXCL9 / Creatinine)**		1,598	108 (6.76)	2.107 (1.691–2.625)	**< 0.001**	173 (10.83)	1.514 (1.226–1.870)	**< 0.001**	46 (2.88)	2.912 (2.168–3.913)	**< 0.001**	72 (4.51)	2.744 (1.908–3.945)	**< 0.001**

RRR: relative risk ratio; MVI: AMR-spectrum microvascular phenotypes; TCMR: TCMR-spectrum tubulointerstitial inflammation; MVI/TCMR: mixed microvascular and T cell–mediated inflammation; BKV: viral nephropathy

1. SUPPLEMENTARY FIGURES

Supplementary Figure 1: Association of uCXCL9:Cr with the activity index

This figure represents the median of uCXCL9:Cr according to the activity index defined as the sum of the following Banff scores: t, i, v, g, ptc, C4d. The activity index ranges from 0 and up to 17, with higher values indicating more severe lesions (C4d is considered as a binary variable as “presence” or “absence”). The lesions were defined according to Banff 2022 classification. The T bars indicate standard errors.

Panel A. Development cohort

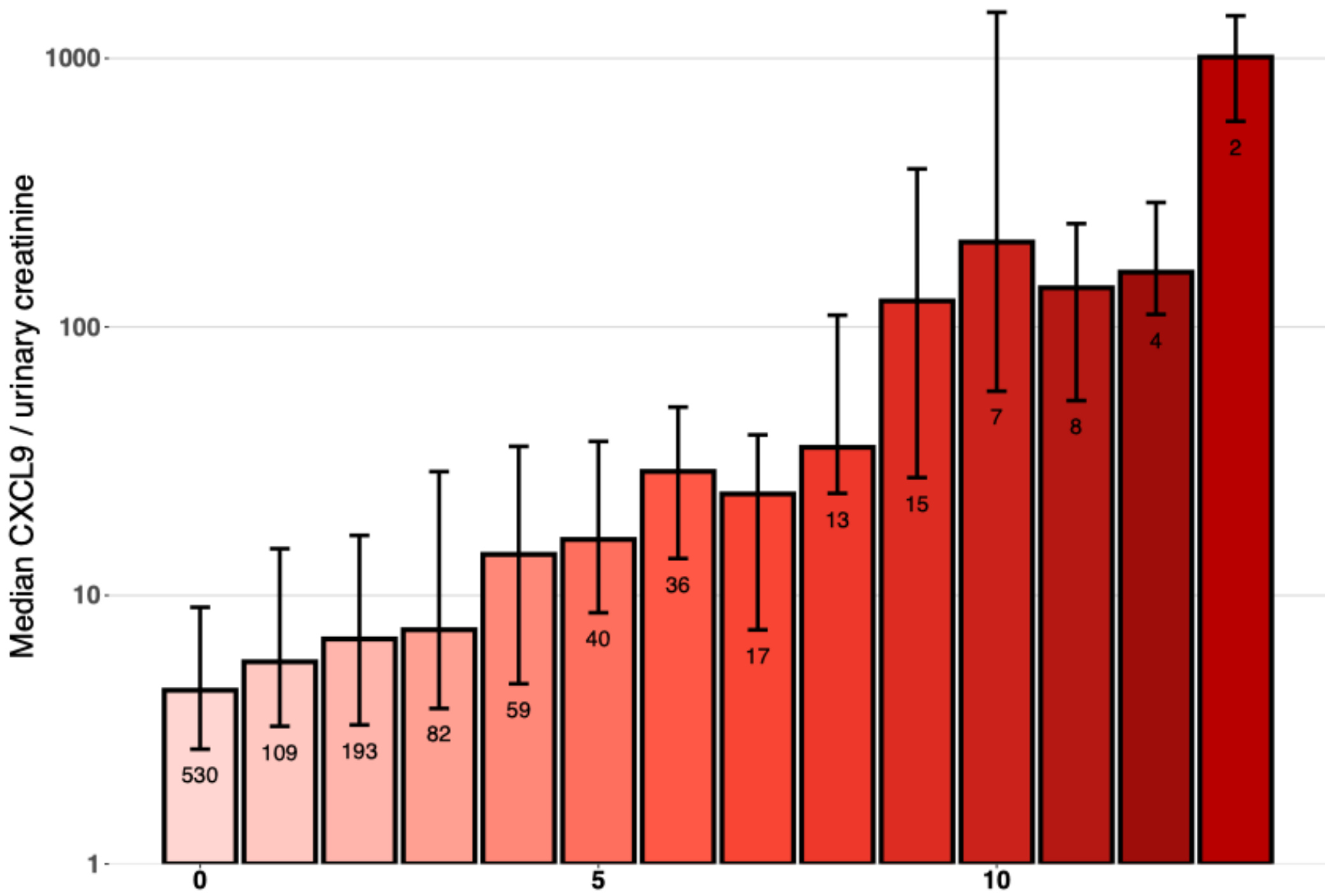

Panel B. Validation cohort

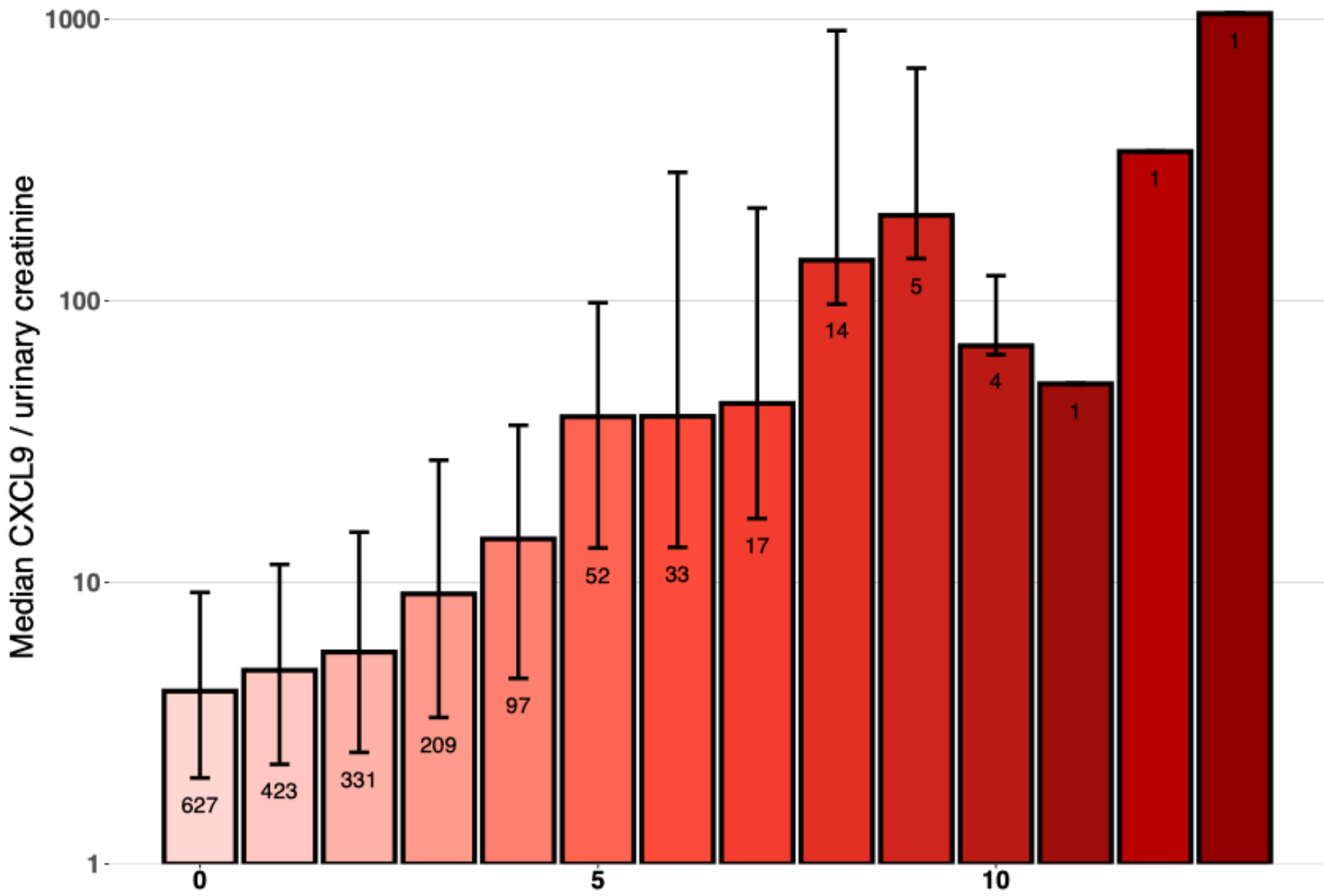

Supplementary Figure 2: Association of uCXCL9:Cr with interstitial fibrosis, tubular atrophy, vascular fibrous intimal thickening and arteriolar hyalinosis

This figure represents the median of uCXCL9:Cr according to the Banff scores of interstitial fibrosis (ci Banff score), tubular atrophy (ct Banff score), vascular fibrous intimal thickening (cv Banff score) and arteriolar hyalinosis (ah Banff score). Each of these scores ranges from 0 to 3, with higher values indicating more severe lesions. The lesions were defined according to Banff 2022 classification. The T bars indicate standard errors. Due to many missing data and the scarcity of high-grade lesion, the mesangial matrix expansion (mm Banff score) variable has been excluded (N=1,375, 42.50%).

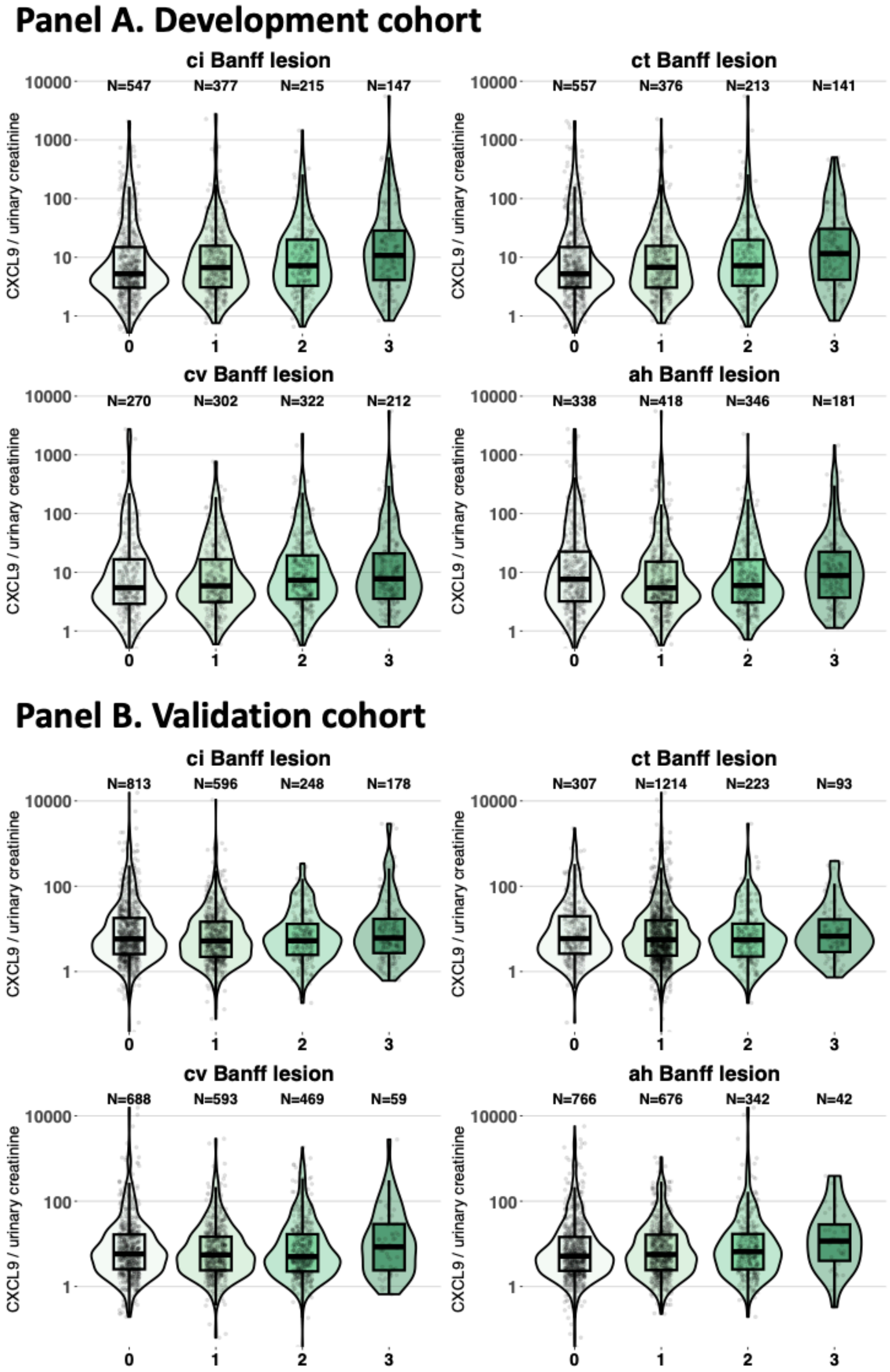

Supplementary Figure 3: Association of uCXCL9:Cr with the chronicity index

This figure represents the median of uCXCL9:Cr according to the chronicity index defined as the sum of the following Banff scores: ci, ct, cv and 2cg. The activity index ranges from 0 to 15, with higher values indicating more severe lesions. The lesions were defined according to Banff 2022 classification. The T bars indicate standard errors.

Panel A. Development cohort

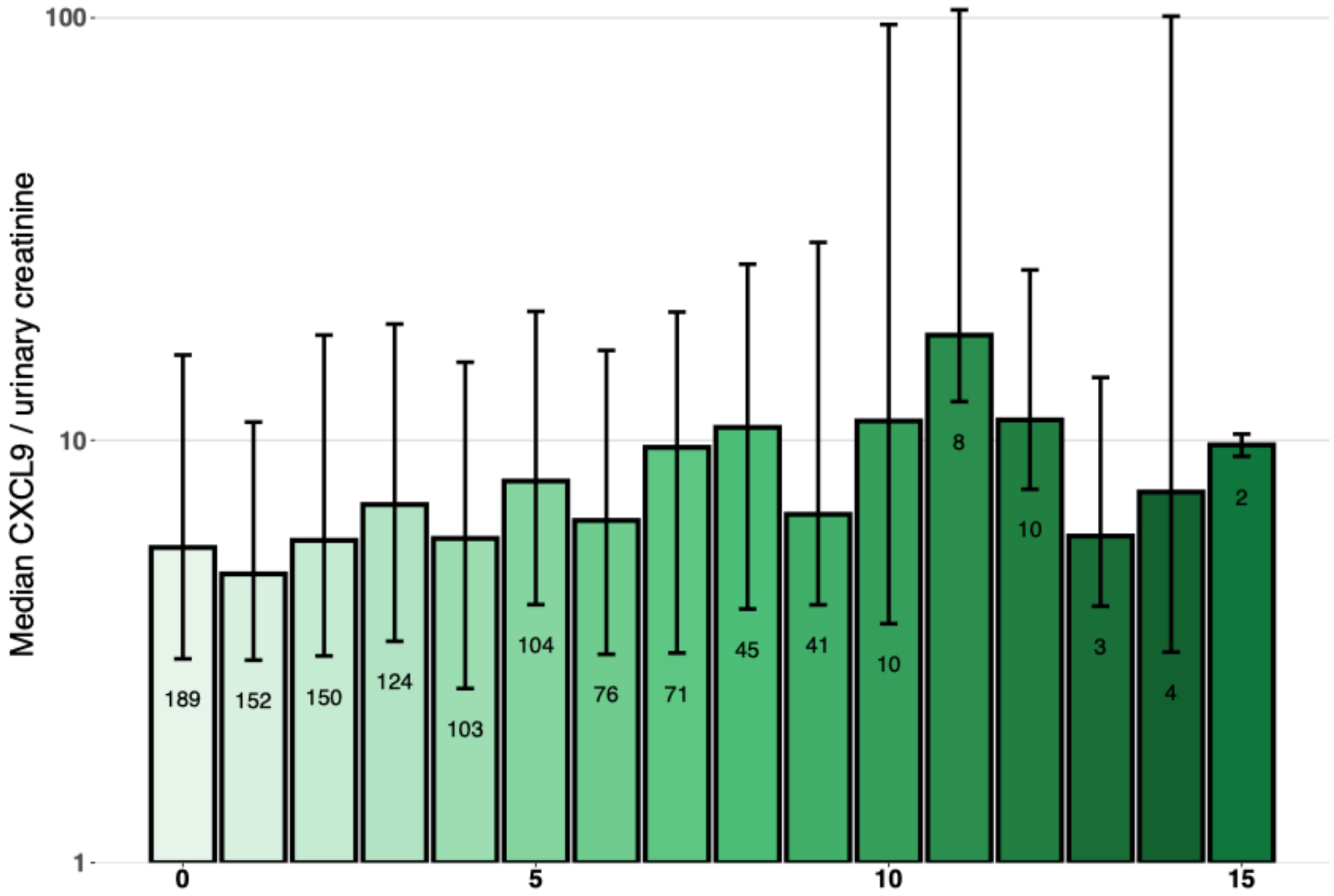

Panel B. Validation cohort

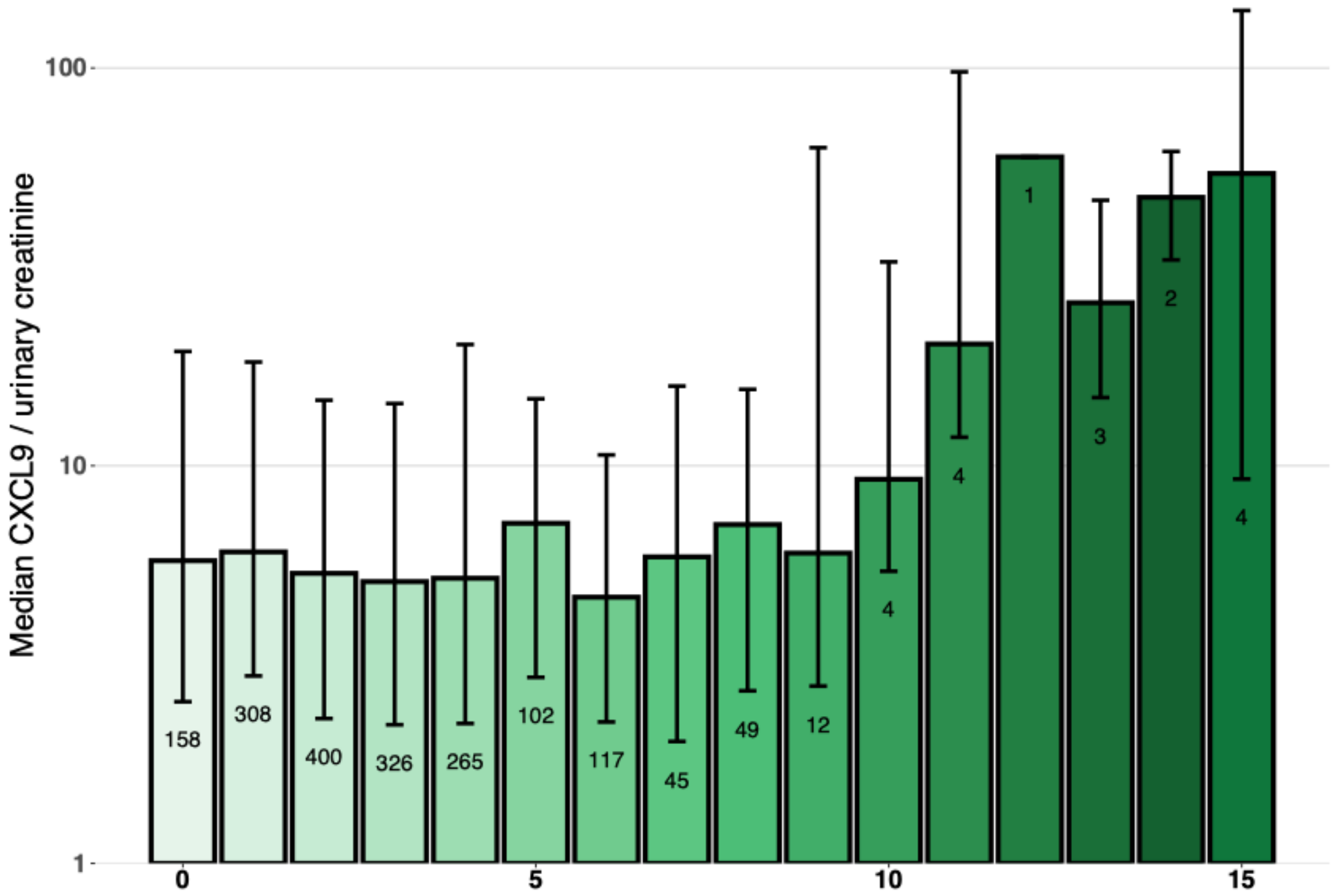

Supplementary Figure 4: Bootstrap multinomial LASSO feature selection

The variables included in the multivariate model were selected using a multinomial LASSO applied across 1,000 bootstrap resamples, retaining predictors with a minimum selection frequency of 90% (red dotted line). This procedure identifies stable predictors (i.e., variables consistently selected across repeated perturbations of the dataset) thereby reducing the risk of overfitting. The dotted golden line delineates the 80% selection threshold.

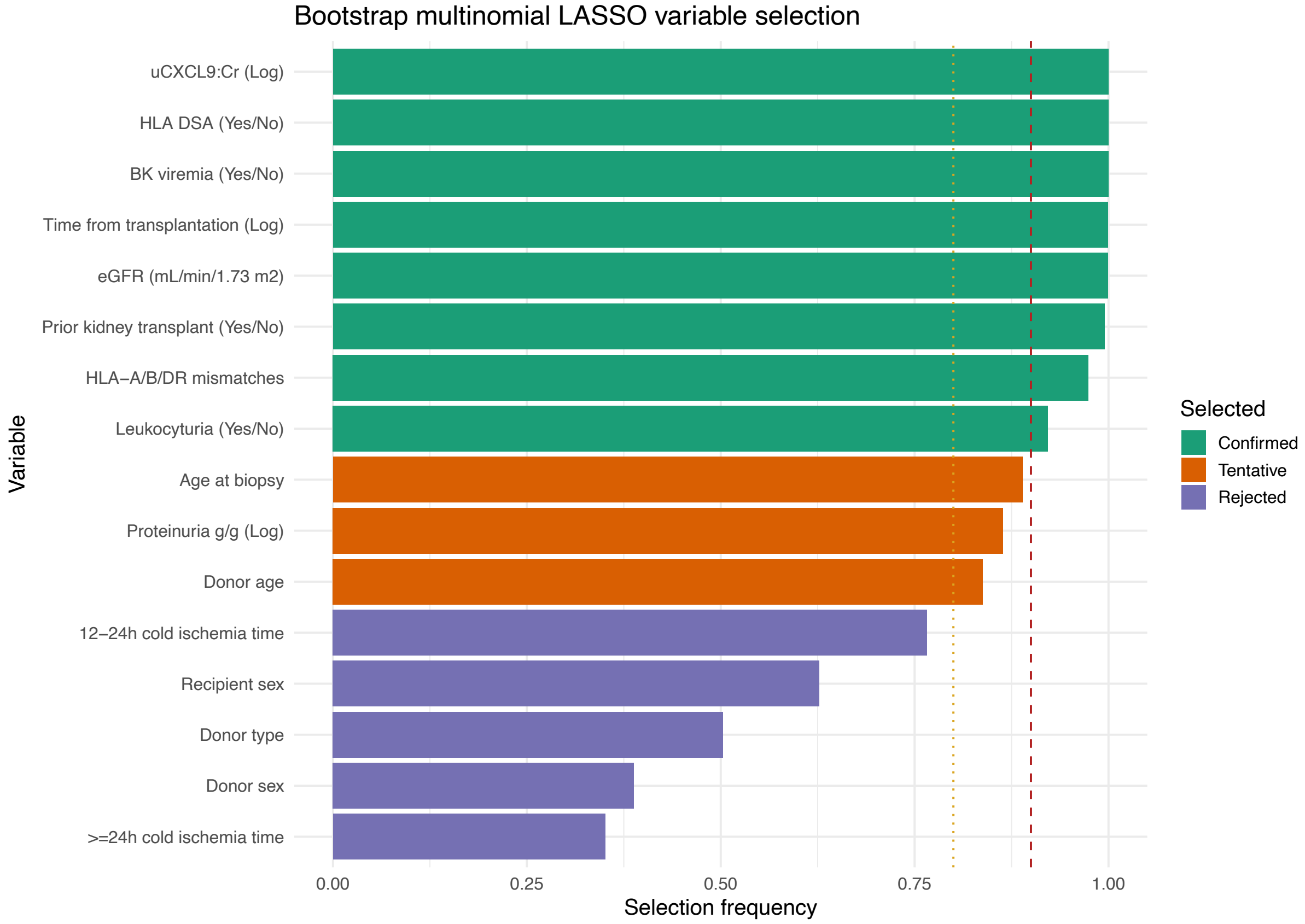

Supplementary Figure 5: Consistent incremental multiclass discrimination conferred by uCXCL9:Cr across single-variable models in the development and in the validation cohort. Pairwise comparisons between single-variable reference models and corresponding models augmented with urinary CXCL9:Cr. For each clinical or immunological variable, the addition of CXCL9:Cr resulted in a consistent and statistically significant improvement in multiclass discrimination, as quantified by the Hand and Till AUC (all $p < 0.001$). Top panel corresponds to the development cohort and bottom panel to the validation cohort.

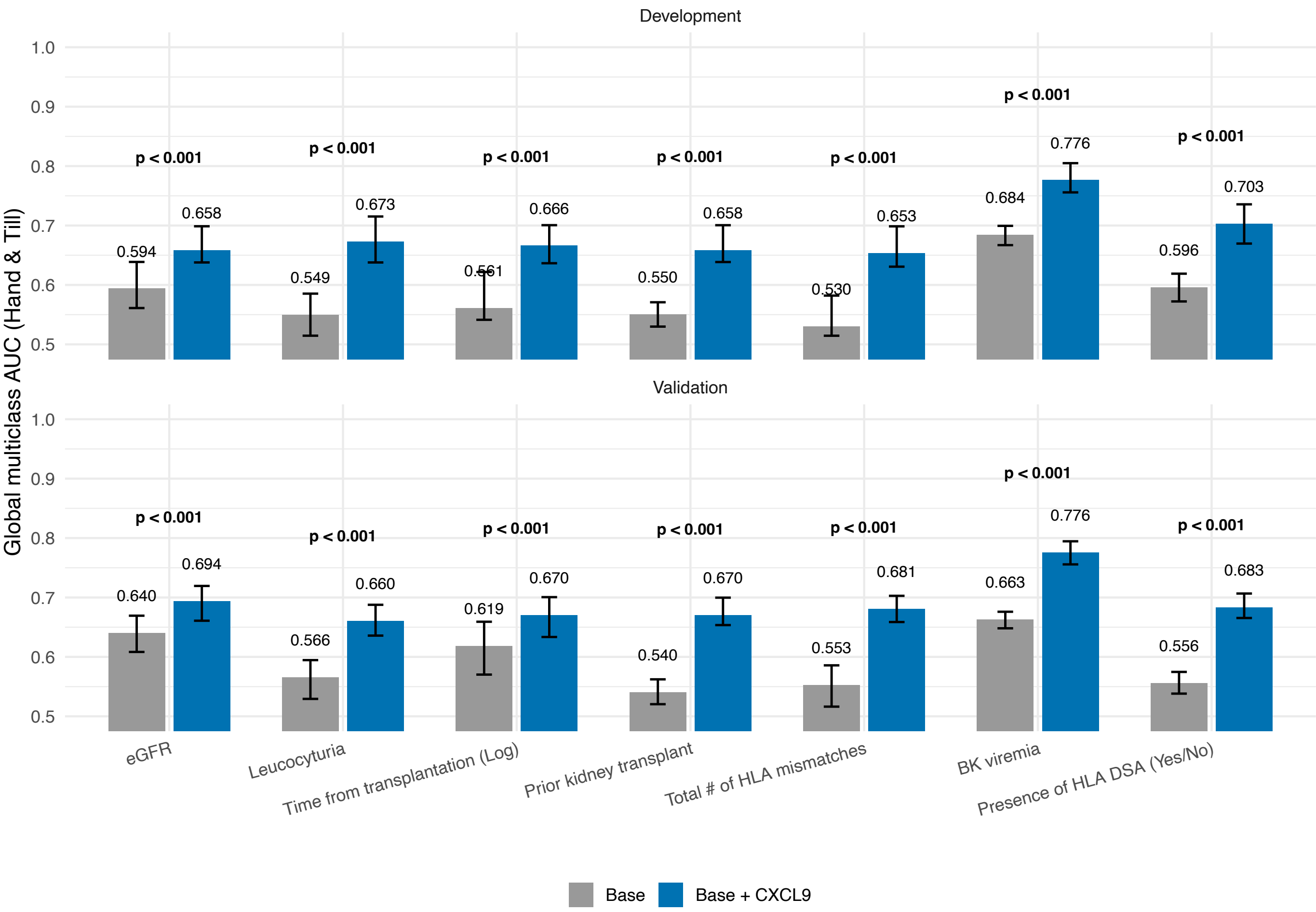

Supplementary Figure 6: Multiclass calibration curves in the development and validation cohorts
Calibration was evaluated per diagnostic class by grouping predicted probabilities into deciles and, within each bin, comparing the mean predicted probability with the observed event frequency. For each class of the multinomial outcome, calibration curves were generated by plotting observed versus predicted risks alongside the identity line, using predictions from both the development (**A**) and validation (**B**) cohorts.

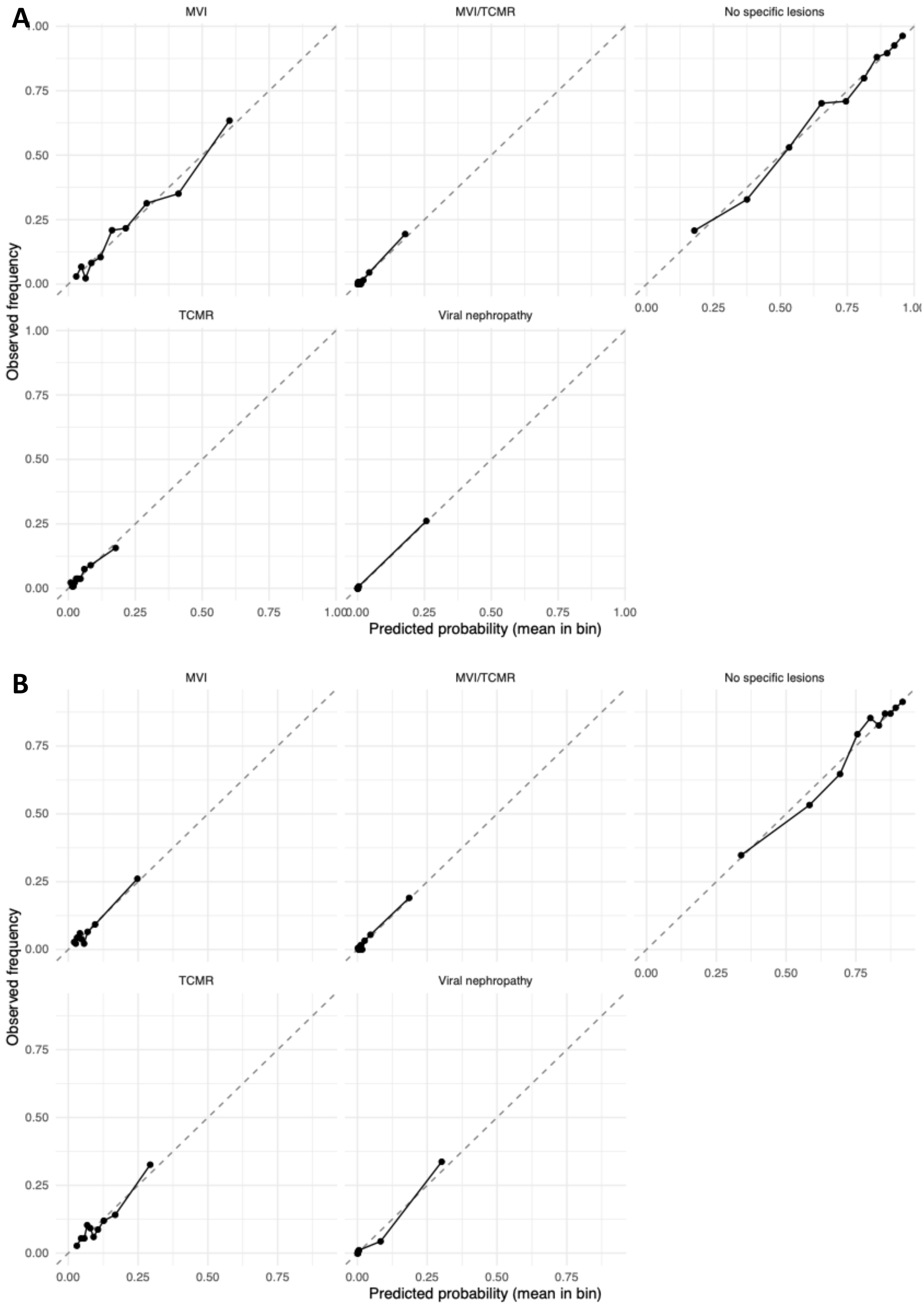

Supplementary Figure 7: Decision curve-derived reduction in unnecessary biopsies with uCXCL9:Cr
Reduction in unnecessary biopsies associated with the addition of uCXCL9:Cr to the clinical model across a range of decision thresholds. The figures shows the net reduction in unnecessary biopsies per 100 patients, derived from decision curve analysis. Positive values indicate fewer biopsies compared with the clinical model alone.

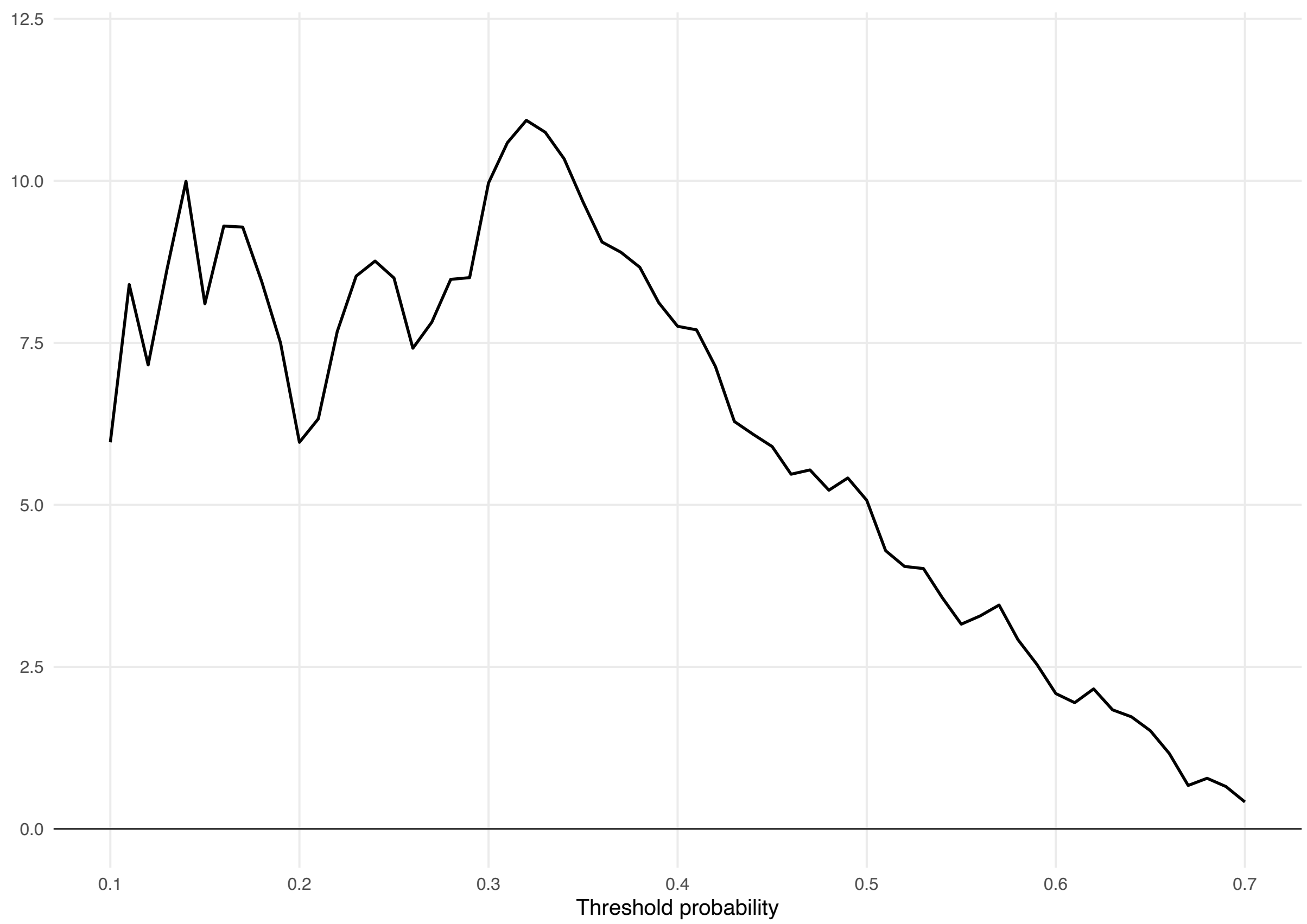

Supplementary Figure 8: AMR/MVI index distributions for high vs low chronicity index in the validation cohort

A slight increase in uCXCL9:Cr was observed in samples with high chronicity indices (≥10) in the validation cohort; this association was evaluated in relation to the distribution of AMR/MVI, given that the chronicity index is largely driven by the Banff cg score.

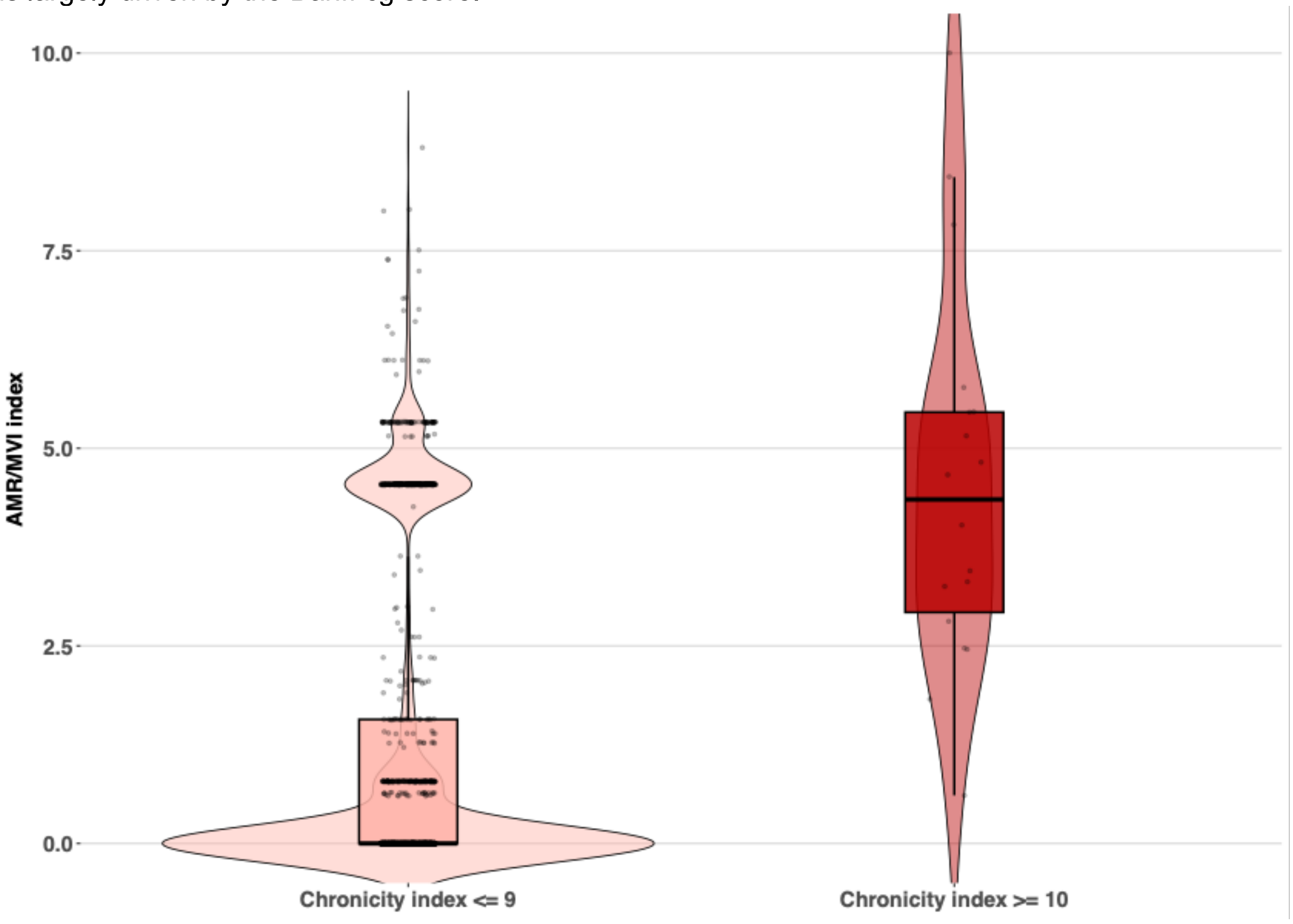

Supplementary Figure 9: Decision curve analysis of the clinical models with or without uCXCL9:Cr in the validation cohort

Decision curve analysis showing the net benefit of the clinical model alone versus the clinical model including urinary CXCL9 across threshold probabilities in the development cohort. The model incorporating CXCL9 provides a consistently higher net benefit between 0.10 and 0.65, the range in which biopsy decisions are typically uncertain. In this interval, CXCL9 improves identification of biopsies likely to show intragraft activity while reducing unnecessary procedures compared with the clinical model alone, as well as the “biopsy-all” and “biopsy-none” strategies.

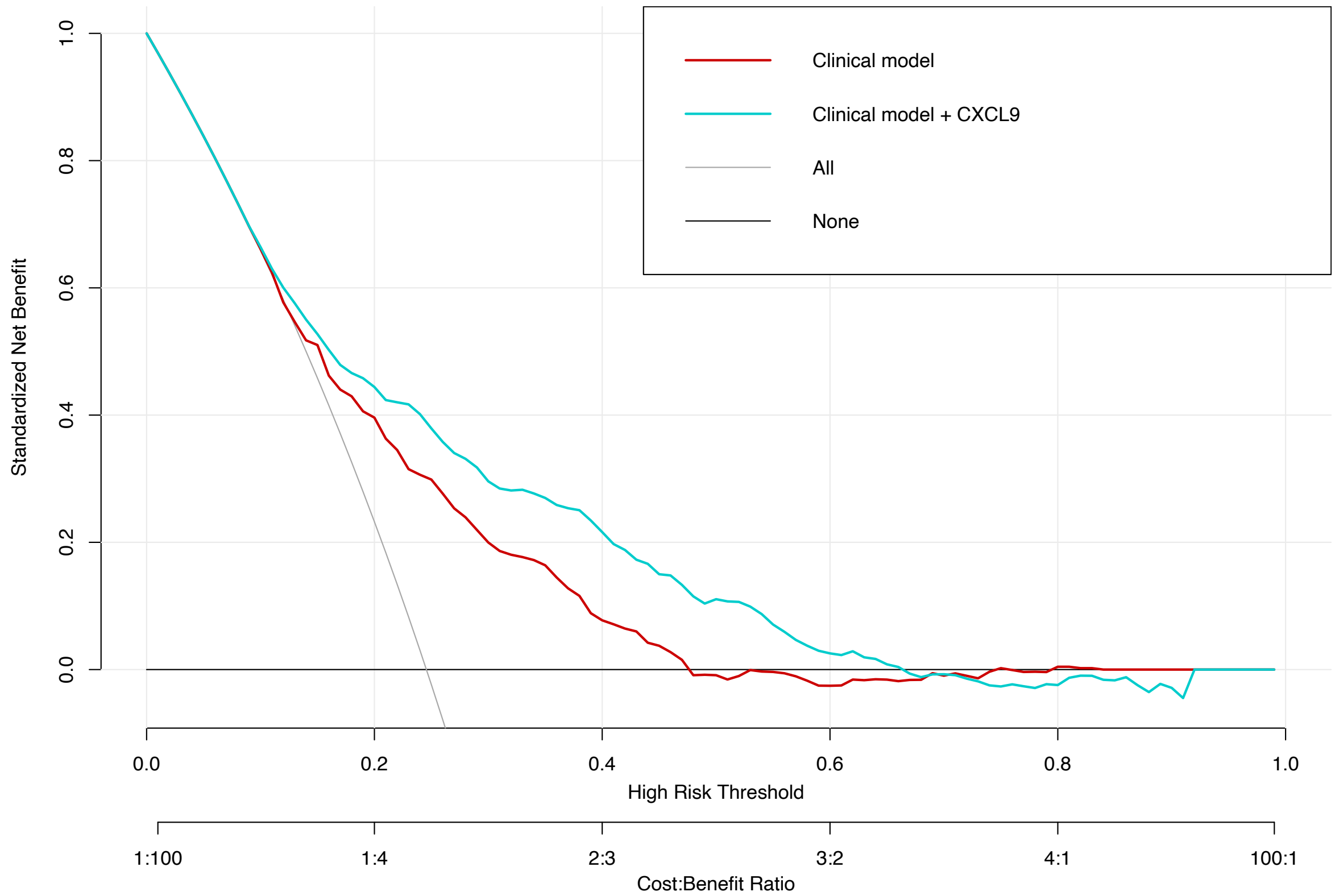

Supplementary Figure 10: Performances of models with uCXCL9:Cr and without uCXCL9:Cr in predicting kidney allograft biopsy lesions (complete case analysis).
Left panel represents the added-value of CXCL9:Cr in the development cohort, while the left panel represents the added-value in the validation cohort

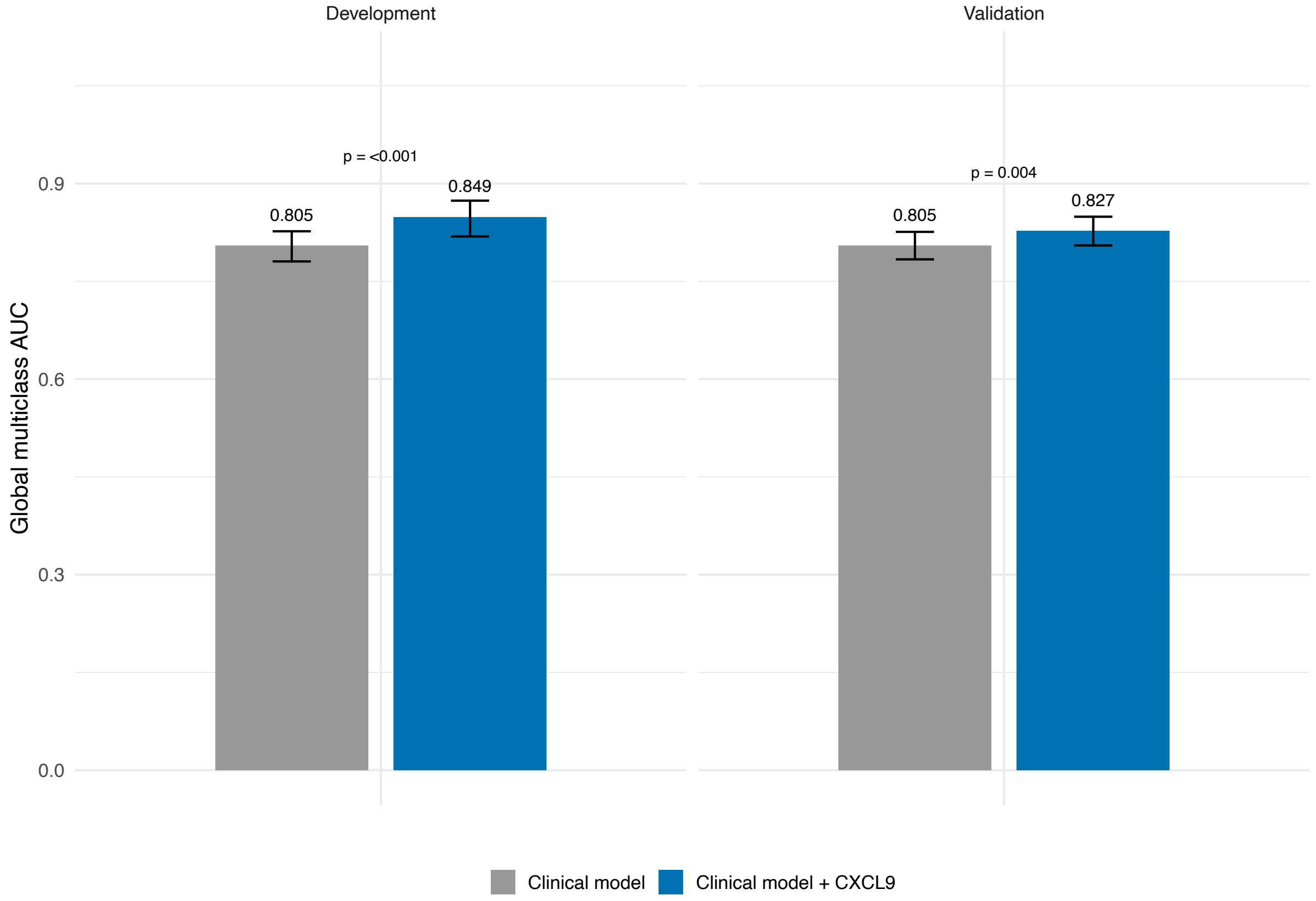

Section & Topic	No	Item	Reported on page #
**TITLE OR ABSTRACT**			
	**1**	Identification as a study of diagnostic accuracy using at least one measure of accuracy (such as sensitivity, specificity, predictive values, or AUC)	Page 2
**ABSTRACT**			
	**2**	Structured summary of study design, methods, results, and conclusions (for specific guidance, see STARD for Abstracts)	Page 2
**INTRODUCTION**			
	**3**	Scientific and clinical background, including the intended use and clinical role of the index test	Page 3
	**4**	Study objectives and hypotheses	Page 4
**METHODS**			
Study design	**5**	Whether data collection was planned before the index test and reference standard were performed (prospective study) or after (retrospective study)	Page 5
Participants	**6**	Eligibility criteria	Page 5
	**7**	On what basis potentially eligible participants were identified (such as symptoms, results from previous tests, inclusion in registry)	Page 5
	**8**	Where and when potentially eligible participants were identified (setting, location and dates)	Page 5
	**9**	Whether participants formed a consecutive, random or convenience series	Page 5
Test methods	**10a**	Index test, in sufficient detail to allow replication	Page 7-9
	**10b**	Reference standard, in sufficient detail to allow replication	Page 8
	**11**	Rationale for choosing the reference standard (if alternatives exist)	Page 8
	**12a**	Definition of and rationale for test positivity cut-offs or result categories of the index test, distinguishing pre-specified from exploratory	Page 8
	**12b**	Definition of and rationale for test positivity cut-offs or result categories of the reference standard, distinguishing pre-specified from exploratory	Page 8
	**13a**	Whether clinical information and reference standard results were available to the performers/readers of the index test	Pages 8
	**13b**	Whether clinical information and index test results were available to the assessors of the reference standard	n/a
Analysis	**14**	Methods for estimating or comparing measures of diagnostic accuracy	Pages 8
	**15**	How indeterminate index test or reference standard results were handled	n/a
	**16**	How missing data on the index test and reference standard were handled	Page 8
	**17**	Any analyses of variability in diagnostic accuracy, distinguishing pre-specified from exploratory	-
	**18**	Intended sample size and how it was determined	-
**RESULTS**			
Participants	**19**	Flow of participants, using a diagram	-
	**20**	Baseline demographic and clinical characteristics of participants	Page 10
	**21a**	Distribution of severity of disease in those with the target condition	Page 10
	**21b**	Distribution of alternative diagnoses in those without the target condition	n/a
	**22**	Time interval and any clinical interventions between index test and reference standard	Page 11-12
Test results	**23**	Cross tabulation of the index test results (or their distribution) by the results of the reference standard	Pages 11- 12
	**24**	Estimates of diagnostic accuracy and their precision (such as 95% confidence intervals)	Page 12-14
	**25**	Any adverse events from performing the index test or the reference standard	-
**DISCUSSION**			
	**26**	Study limitations, including sources of potential bias, statistical uncertainty, and generalisability	Page 19-20
	**27**	Implications for practice, including the intended use and clinical role of the index test	Pages 18-19
**OTHER INFORMATION**			
	**28**	Registration number and name of registry	-
	**29**	Where the full study protocol can be accessed	-
	**30**	Sources of funding and other support; role of funders	Page 21

cc BY SA

STARD 2015

AIM

STARD stands for “Standards for Reporting Diagnostic accuracy studies”. This list of items was developed to contribute to the completeness and transparency of reporting of diagnostic accuracy studies. Authors can use the list to write informative study reports. Editors and peer-reviewers can use it to evaluate whether the information has been included in manuscripts submitted for publication.

EXPLANATION

A **diagnostic accuracy study** evaluates the ability of one or more medical tests to correctly classify study participants as having a **target condition.** This can be a disease, a disease stage, response or benefit from therapy, or an event or condition in the future. A medical test can be an imaging procedure, a laboratory test, elements from history and physical examination, a combination of these, or any other method for collecting information about the current health status of a patient.

The test whose accuracy is evaluated is called **index test.** A study can evaluate the accuracy of one or more index tests. Evaluating the ability of a medical test to correctly classify patients is typically done by comparing the distribution of the index test results with those of the **reference standard**. The reference standard is the best available method for establishing the presence or absence of the target condition. An accuracy study can rely on one or more reference standards.

If test results are categorized as either positive or negative, the cross tabulation of the index test results against those of the reference standard can be used to estimate the **sensitivity** of the index test (the proportion of participants *with* the target condition who have a positive index test), and its **specificity** (the proportion *without* the target condition who have a negative index test). From this cross tabulation (sometimes referred to as the contingency or “2x2” table), several other accuracy statistics can be estimated, such as the positive and negative **predictive values** of the test. Confidence intervals around estimates of accuracy can then be calculated to quantify the statistical **precision** of the measurements.

If the index test results can take more than two values, categorization of test results as positive or negative requires a **test positivity cut-off**. When multiple such cut-offs can be defined, authors can report a receiver operating characteristic (ROC) curve which graphically represents the combination of sensitivity and specificity for each possible test positivity cut-off. The **area under the ROC curve** informs in a single numerical value about the overall diagnostic accuracy of the index test.

The **intended use** of a medical test can be diagnosis, screening, staging, monitoring, surveillance, prediction or prognosis. The **clinical role** of a test explains its position relative to existing tests in the clinical pathway. A replacement test, for example, replaces an existing test. A triage test is used before an existing test; an add-on test is used after an existing test.

Besides diagnostic accuracy, several other outcomes and statistics may be relevant in the evaluation of medical tests. Medical tests can also be used to classify patients for purposes other than diagnosis, such as staging or prognosis. The STARD list was not explicitly developed for these other outcomes, statistics, and study types, although most STARD items would still apply.

DEVELOPMENT

This STARD list was released in 2015. The 30 items were identified by an international expert group of methodologists, researchers, and editors. The guiding principle in the development of STARD was to select items that, when reported, would help readers to judge the potential for bias in the study, to appraise the applicability of the study findings and the validity of conclusions and recommendations. The list represents an update of the first version, which was published in 2003.

More information can be found on http://www.equator-network.org/reporting-guidelines/stard.

CC BY SA

TRIPOD Checklist: Prediction Model Development

Section/Topic	Item	Checklist Item	Page
**Title and abstract**			
Title	1	Identify the study as developing and/or validating a multivariable prediction model, the target population, and the outcome to be predicted.	1
Abstract	2	Provide a summary of objectives, study design, setting, participants, sample size, predictors, outcome, statistical analysis, results, and conclusions.	2
**Introduction**			
Background and objectives	3a	Explain the medical context (including whether diagnostic or prognostic) and rationale for developing or validating the multivariable prediction model, including references to existing models.	3-4
	3b	Specify the objectives, including whether the study describes the development or validation of the model or both.	4
**Methods**			
Source of data	4a	Describe the study design or source of data (e.g., randomized trial, cohort, or registry data), separately for the development and validation data sets, if applicable.	5
	4b	Specify the key study dates, including start of accrual; end of accrual; and, if applicable, end of follow-up.	5
Participants	5a	Specify key elements of the study setting (e.g., primary care, secondary care, general population) including number and location of centres.	5
	5b	Describe eligibility criteria for participants.	5
	5c	Give details of treatments received, if relevant.	n/a
Outcome	6a	Clearly define the outcome that is predicted by the prediction model, including how and when assessed.	6-7
	6b	Report any actions to blind assessment of the outcome to be predicted.	n/a
Predictors	7a	Clearly define all predictors used in developing or validating the multivariable prediction model, including how and when they were measured.	8-9
	7b	Report any actions to blind assessment of predictors for the outcome and other predictors.	n/a
Sample size	8	Explain how the study size was arrived at.	8
Missing data	9	Describe how missing data were handled (e.g., complete-case analysis, single imputation, multiple imputation) with details of any imputation method.	8
Statistical analysis methods	10a	Describe how predictors were handled in the analyses.	7-8
	10b	Specify type of model, all model-building procedures (including any predictor selection), and method for internal validation.	7-8
	10d	Specify all measures used to assess model performance and, if relevant, to compare multiple models.	7-8
Risk groups	11	Provide details on how risk groups were created, if done.	n/a
**Results**			
Participants	13a	Describe the flow of participants through the study, including the number of participants with and without the outcome and, if applicable, a summary of the follow-up time. A diagram may be helpful.	10
	13b	Describe the characteristics of the participants (basic demographics, clinical features, available predictors), including the number of participants with missing data for predictors and outcome.	10-11
Model development	14a	Specify the number of participants and outcome events in each analysis.	10-11
	14b	If done, report the unadjusted association between each candidate predictor and outcome.	9- 10
Model specification	15a	Present the full prediction model to allow predictions for individuals (i.e., all regression coefficients, and model intercept or baseline survival at a given time point).	12
	15b	Explain how to the use the prediction model.	18-19
Model performance	16	Report performance measures (with CIs) for the prediction model.	12-14
**Discussion**			
Limitations	18	Discuss any limitations of the study (such as nonrepresentative sample, few events per predictor, missing data).	19-20
Interpretation	19b	Give an overall interpretation of the results, considering objectives, limitations, and results from similar studies, and other relevant evidence.	19-21
Implications	20	Discuss the potential clinical use of the model and implications for future research.	18-19
**Other information**			
Supplementary information	21	Provide information about the availability of supplementary resources, such as study protocol, Web calculator, and data sets.	23
Funding	22	Give the source of funding and the role of the funders for the present study.	21

We recommend using the TRIPOD Checklist in conjunction with the TRIPOD Explanation and Elaboration document.
